# The *Carpiodes* Conundrum: Molecular Hypothesis Testing Informs Conservation Applications for Carpsuckers (Catostomidae: *Carpiodes*) in Texas and Beyond

**DOI:** 10.1002/ece3.72543

**Published:** 2025-11-23

**Authors:** H. C. Roberts, P. T. Bean, K. D. Keith, K. W. Conway, J. S. Perkin

**Affiliations:** ^1^ Department of Ecology and Conservation Biology Texas A&M University College Station Texas USA; ^2^ Heart of the Hills Fisheries Science Center, Inland Fisheries Division Texas Parks and Wildlife Department Mountain Home Texas USA

**Keywords:** biogeography, cryptic diversity, hybridization, taxonomy

## Abstract

Sufficient taxonomic understanding is critical for biodiversity conservation. This is particularly relevant among freshwater fishes, where cryptic undescribed species cause difficulties for promoting conservation efforts. Catostomidae (i.e., suckers) is a family of freshwater fishes with cryptic diversity and biological traits that make them difficult to classify taxonomically. Among suckers, the Carpsuckers ( *Carpiodes carpio* , *Carpiodes cyprinus* , *Carpiodes velifer* ) possess uncertain taxonomic classifications and cryptic diversity despite a rich history of research. Within *Carpiodes*, uniquely slender‐bodied populations occurring in Western Gulf of Mexico drainages suggest potential for an undescribed species. Originally collected in the Llano River, tributary to the Texas Colorado River, Llano River Carpsucker are morphologically similar to *C. cyprinus* . Our study explores how historical biogeographic scenarios may have led to lineage diversification of Llano River Carpsucker. We test competing molecular hypotheses (i.e., Native Endemic Species Hypothesis, Native Lineage Hypothesis) to explain the native origin of Llano River Carpsucker and further assess whether the taxon is nonnative *C. cyprinus* (i.e., Species Introduction Hypothesis), each carrying vastly different conservation and management implications. Additionally, we assessed phylogenetic relationships across the entire genus *Carpiodes*. Phylogenetic analyses recovered divergent lineages of *C. cyprinus* in Eastern Gulf of Mexico drainages, suggesting the presence of cryptic undescribed species. Llano River Carpsucker specimens were resolved in unique lineages relative to *C. cyprinus* , with mitochondrial haplotypes closely related to Mississippi *C. cyprinus* (*p*‐distance < 0.005). Our study suggests Llano River Carpsucker represent native *C. cyprinus* , supporting our Native Lineage Hypothesis. We further provide evidence that *C. cyprinus* readily hybridizes with *C. carpio* , resulting in mitochondrial introgression across much of their distribution. Lastly, we provide recommendations to promote conservation efforts and discuss further research directions to understand deeper evolutionary and environmental mechanisms behind morphologically and genetically unique *C. cyprinus* inhabiting Western Gulf of Mexico drainages of Texas.

## Graphical abstract

Our study investigates both distribution‐wide and within‐Texas genetic patterns of a group of fishes in need of greater taxonomic research (Catostomidae: *Carpiodes*). We recovered lineages of *Carpiodes* in the southeastern United States that are likely cryptic undescribed species and concurrently identify the native and taxonomic status of a uniquely shaped *Carpiodes* predominantly inhabiting the Texas Hill Country ecoregion.

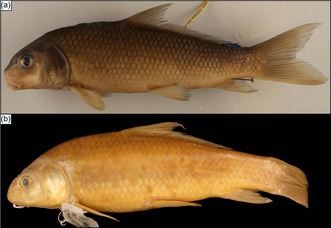

## 1 Introduction

When taxa lack recognition, they often cannot be conserved appropriately and ultimately may become extinct (Duncan and Lockwood 2001; Mace 2004). This is relevant for the ray‐finned fishes (Class Actinopterygii) as they exhibit the highest diversity among vertebrates and are in need of further taxonomic research (Lundberg et al. 2000; Nelson et al. 2016). Of special concern are freshwater fishes, as their habitats intersect terrestrial landscapes making them directly susceptible to anthropogenic pressures (Dudgeon et al. 2006; Seehausen and Wagner 2014). Many freshwater fishes are considered species complexes, often because taxa are morphologically similar (i.e., cryptically diverse) despite being genetically distinct (e.g., Melo et al. 2016). Conservation efforts can be misled when cryptic taxa are present as unrecognized species may have different biological or ecological requirements not considered when treating a complex as a single species (Hending 2025). Therefore, further taxonomic investigation is needed to facilitate biodiversity conservation of freshwater fishes that accounts for cryptic species diversity.

A family of freshwater fishes in need of further taxonomic investigation is the Holarctic Catostomidae (i.e., suckers). Currently, 87 species of suckers are recognized across 15 genera (Fricke et al. 2025). Classification of suckers is often problematic as they are tetraploids and are prone to hybridization (Bart Jr. et al. 2010; Unmack et al. 2014; Yang et al. 2024). Additionally, many recognized suckers likely represent species complexes harboring cryptic diversity (Pérez‐Rodríguez et al. 2016; Hunt et al. 2021). Suckers have also been introduced outside their native range and are known to detrimentally hybridize with native congeners (McDonald et al. 2008; Sweet and Hubert 2010). Considering the above, taxonomic and genetic investigation of suckers is essential for their conservation.

**FIGURE 1:**
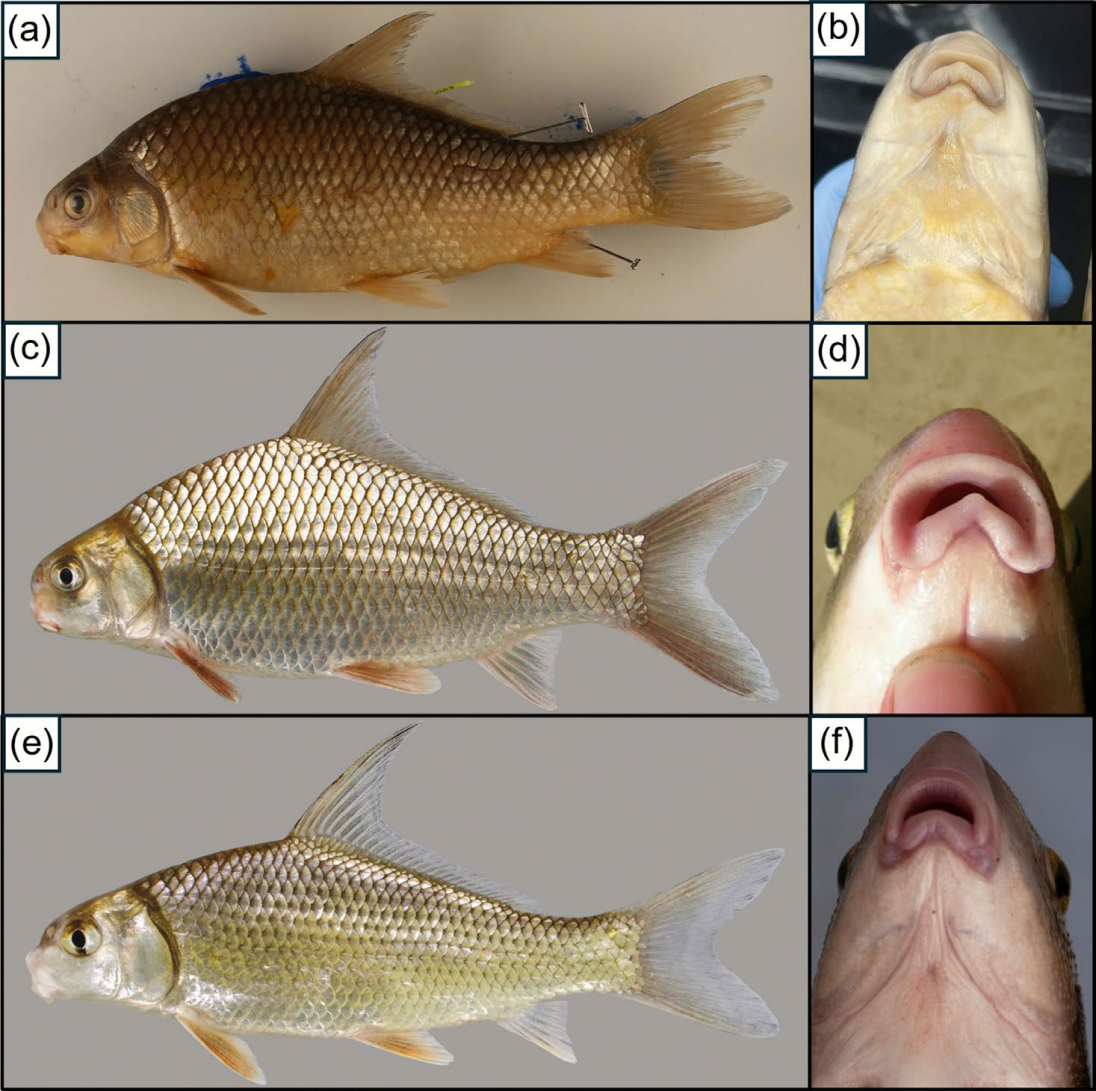
Morphological descriptions of recognized *Carpiodes* species. (a) *Carpiodes carpio* with 35 lateral‐line scales (range for species = 34–37) and (b) a lower lip protuberance. *Carpiodes carpio* inhabiting the Rio Grande and coastal drainages of northeastern Mexico are known to possess a weakly developed or absent lower lip protuberance. (c) *Carpiodes velifer* with 35 lateral‐line scales (range for species = 33–36), an anterior dorsal‐fin ray reaching the back of the dorsal‐fin, and (d) a lower lip protuberance. (e) *Carpiodes cyprinus* with 37 lateral‐line scales (range for species = 37–40) and (f) with no lower lip protuberance. Morphological information for *C. carpio* is from Hubbs and Black (1940), and Pflieger (1997), while *C. cyprinus* and *C. velifer* were obtained from (Hubbs et al. 2004). Photographs in (a, b) are of TCWC 20629.01, (c) of UF 238170 by Zach Randall, Florida Museum of Natural History, (d) by Nate Tessler, (e) of UF 238188 by Zach Randall, Florida Museum of Natural History, and (f) by Scott A. Smith. All photographs were granted permission for use.

**FIGURE 2:**
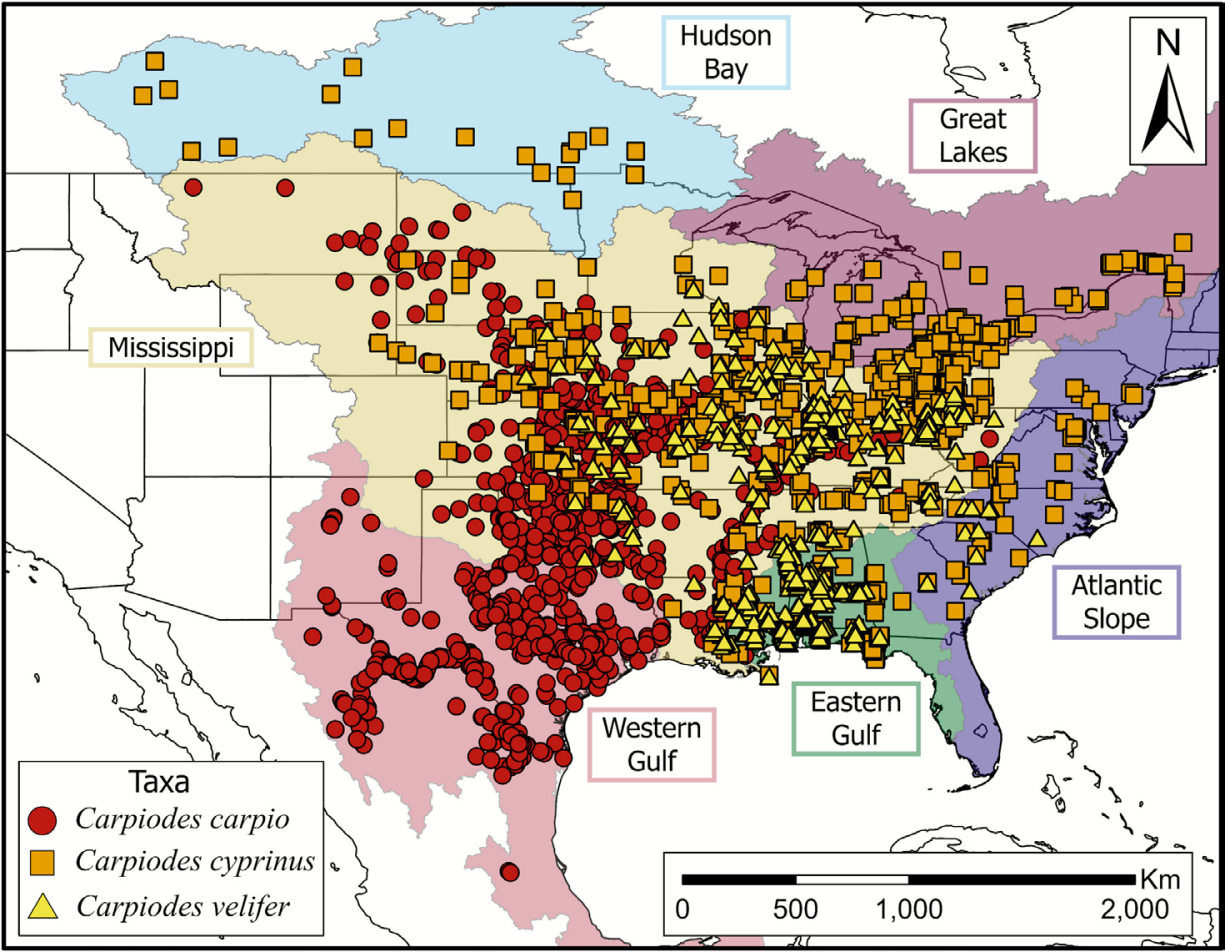
Distribution maps of recognized *Carpiodes* species. Observations are derived from the Global Biodiversity Information Facilities database (GBIF 2024) and represent native distributions following Page and Burr (2011). *Carpiodes* localities intersect Atlantic Slope, Eastern Gulf of Mexico (i.e., Eastern Gulf), Great Lakes, Hudson Bay, Mississippi, and Western Gulf of Mexico (i.e., Western Gulf) basins, described in Table 1. Extent of basins were modified from the North American Atlas Basin Watersheds dataset (Commission for Environmental Cooperation 2010). Symbols denote recognized species where circles are *Carpiodes carpio*, squares are *Carpiodes cyprinus*, and triangles are *Carpiodes velifer*.

Among catostomids, the genus *Carpiodes* (Carpsuckers) currently includes three recognized species ( *Carpiodes carpio* , *Carpiodes velifer* , *Carpiodes cyprinus* ) distinguished based on differences in mouth morphology, meristics, fin measurements, and breeding tubercles (Huntsman 1967; Pflieger 1997; Hubbs et al. 2004). *Carpiodes carpio* (River Carpsucker) possesses 34–37 lateral‐line scales and typically a lower lip protuberance (Figure 1a,b). *Carpiodes velifer* (Highfin Carpsucker) possesses a lower lip protuberance and has 33–36 lateral‐line scales (Figure 1c,d). *Carpiodes velifer* is distinguished from *C. carpio* by possessing an anterior dorsal‐fin ray reaching the posterior fin margin when depressed. *Carpiodes cyprinus* (Quillback) is distinguished from other *Carpiodes* as it possesses 37–40 lateral‐line scales and does not have a lower lip protuberance (Figure 1e,f). Currently, all recognized *Carpiodes* species inhabit the Mississippi basin (Figure 2). *Carpiodes carpio* further inhabits the Western Gulf of Mexico (i.e., Western Gulf) basin to the Rio Grande and south into Mexico. *Carpiodes cyprinus* also inhabits the Atlantic Slope, Eastern Gulf of Mexico (i.e., Eastern Gulf), Great Lakes, and Hudson Bay basins, while *C. velifer* also occupies these basins excluding Hudson Bay (see Table 1 for basin definitions).

**TABLE 1:** Description of basins assessed in this study. Basins represent geographically separated areas across North America and are modified from the North American Atlas Basin Watersheds dataset (Commission for Environmental Cooperation 2010). Each basin is described below. When applicable, river boundaries defining the extent of each basin are also included.

Basin	Description
Atlantic Slope	Drainages entering the Atlantic Ocean from North Labrador (Eclipse River, Canada) south to rivers draining the Florida Everglades (Roberts River)
Eastern Gulf	Drainages entering Gulf of Mexico from central Florida (Peace River) west to the Amite River
Great Lakes	All Great Lakes and drainages ultimately entering the Saint Lawrence River
Hudson Bay	Drainages entering the Nelson River draining into Hudson Bay
Mississippi	Entire Mississippi drainage including major tributaries (e.g., Missouri, Ohio) ultimately draining into to the Gulf of Mexico. Adjacent drainages west to the Calcasieu River
Western Gulf	Drainages west of the Mississippi draining into the Gulf of Mexico. Includes all drainages from the Sabine River west to the Rio Grande and southeastern drainages in Mexico to the Champotón River

While three *Carpiodes* species are currently recognized, the genus has a rich history of taxonomic study. Meek (1904) described *Carpiodes elongatus* as a slender‐bodied taxon inhabiting coastal drainages of northeastern Mexico. *Carpiodes elongatus* was later classified as *Carpiodes carpio elongatus* by Hubbs and Black (1940) who noted the taxon also possesses a weakly developed or absent lower lip protuberance and is present in the Rio Grande drainage. This taxon is currently considered synonymous with *Carpiodes carpio* (Lee et al. 1980; Gilbert 1998). Further east, Hubbs (1930) described *Carpiodes forbesi* as a distinct species inhabiting the Mississippi basin and considered the taxon more elongated than *Carpiodes cyprinus* inhabiting the Great Lakes. *Carpiodes cyprinus* was later divided into two subspecies where Hubbs and Lagler (1947) recognized *C. cyprinus cyprinus* and Trautman (1956) described *C. cyprinus hinei*. Trautman (1956) considered *C. c. hinei* to be of intermediate body depth between *C. c. cyprinus* and *C. forbesi* . As an alternative viewpoint, Bailey and Allum (1962) did not consider these taxa valid and associated their morphological differences with environmental attributes. Currently, all three taxa are synonymous with *C. cyprinus* following Lee et al. (1980) and Gilbert (1998). However, some authors continue to recognize *C. cyprinus* subspecies (Hubbs et al. 2004). Additionally, *Carpiodes* with uncertain taxonomic identification (e.g., *Carpiodes* cf. *cyprinus*) are referenced in the southeastern United States (Coughlan et al. 2007). While certain *Carpiodes* are referred to as junior synonyms to *C. cyprinus* (i.e., *C. forbesi* ), competing philosophies on recognizing *C. cyprinus* subspecies and reference to *C*. cf. *cyprinus* suggest further taxonomic work on the genus is warranted.

**FIGURE 3:**
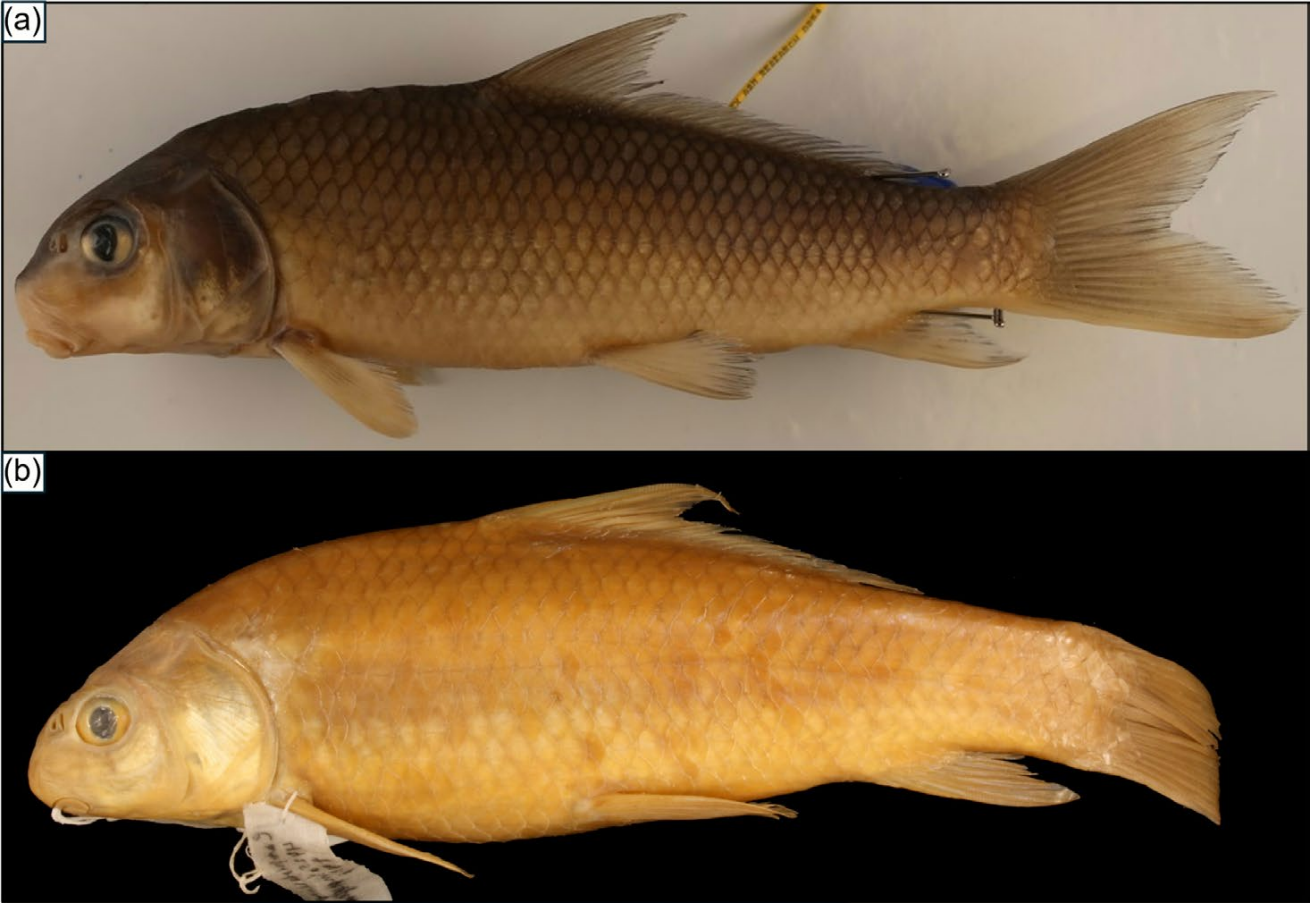
Photographs of additional *Carpiodes* taxa. (a) Llano River Carpsucker (LRCS) with 38 lateral‐line scales (range for specimens in this study = 36–39). Llano River Carpsucker do not possess a lower lip protuberance as in *Carpiodes cyprinus*. (b) *Carpiodes elongatus* holotype with 35 lateral‐line scales. This specimen was collected from the Río San Fernando drainage of Nuevo León, Mexico and was used as the original type specimen in the description of *C. elongatus* by Meek (1904). Hubbs and Black (1940) classified *C. elongatus* as *Carpiodes carpio elongatus* and suggested the taxon has 35–37 lateral‐line scales, possesses a weakly developed or absent lower lip protuberance, and also is present in the Rio Grande. This taxon is now synonymous with *C. carpio* (Lee et al. 1980; Gilbert 1998). Photograph in (a) is of TCWC 20629.01, and (b) of FMNH 4425 used with permission (Field Museum of Natural History ‐ Division of Fishes).

**FIGURE 4:**
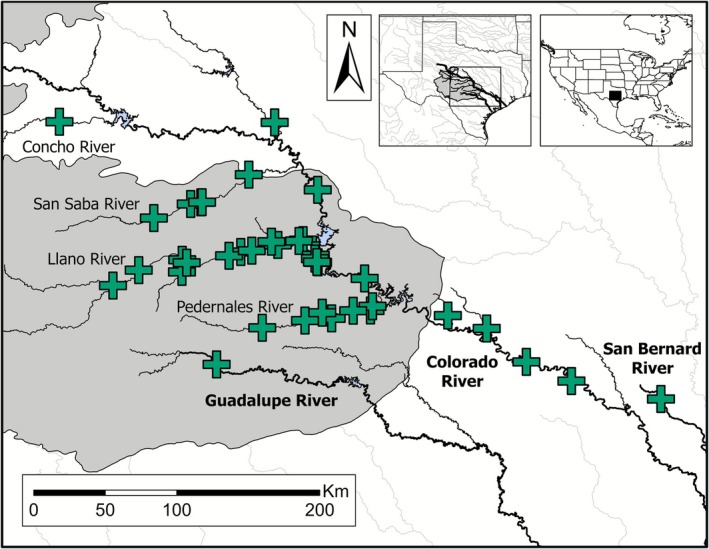
Distribution of Llano River Carpsucker. Crosses display locations where the taxon has been collected across Colorado, Guadalupe, and San Bernard drainages. Principal rivers across drainages are highlighted in bold. Observations stem from collections conducted in this study, confirmed records of the taxon housed at the Fishes of Texas Database (Hendrickson and Cohen 2022), and specimens collected in the San Bernard drainage (Adam Cohen, *Personal communication*). The grey shaded region denotes the Edwards Plateau ecoregion where the taxon is predominantly found.

In Texas, *C. carpio* occurs in sympatry with an undescribed form of *Carpiodes* referred to as Llano River Carpsucker (LRCS) by the Fishes of Texas (FOT) database (Hendrickson and Cohen 2022). The taxon is morphologically similar to *C. cyprinus* as it lacks a lower lip protuberance and has 36–39 lateral‐line scales but is noticeably elongated (Figure 3a). While the slender body of LRCS is similar to Rio Grande *C. carpio* (Figure 3b), Hubbs and Black (1940) describe Rio Grande populations as having 35–37 lateral‐line scales. Considering this, we refer to LRCS as a separate taxon distinct from Rio Grande *C. carpio* . Llano River Carpsucker is predominantly found in Edwards Plateau tributaries of the Texas Colorado River but is also present in Guadalupe and San Bernard drainages (Figure 4). First collected in 1963 along the Llano River, tributary to the Colorado River, records indicate LRCS may be an undescribed species (Hendrickson and Cohen 2022). This is supported by Chen et al. (2010), where morphometric cross‐validation techniques on specimens from Colorado‐Rio Grande drainages suggested slender‐bodied *Carpiodes* were morphologically distinct and may represent a unique species.

**FIGURE 5:**
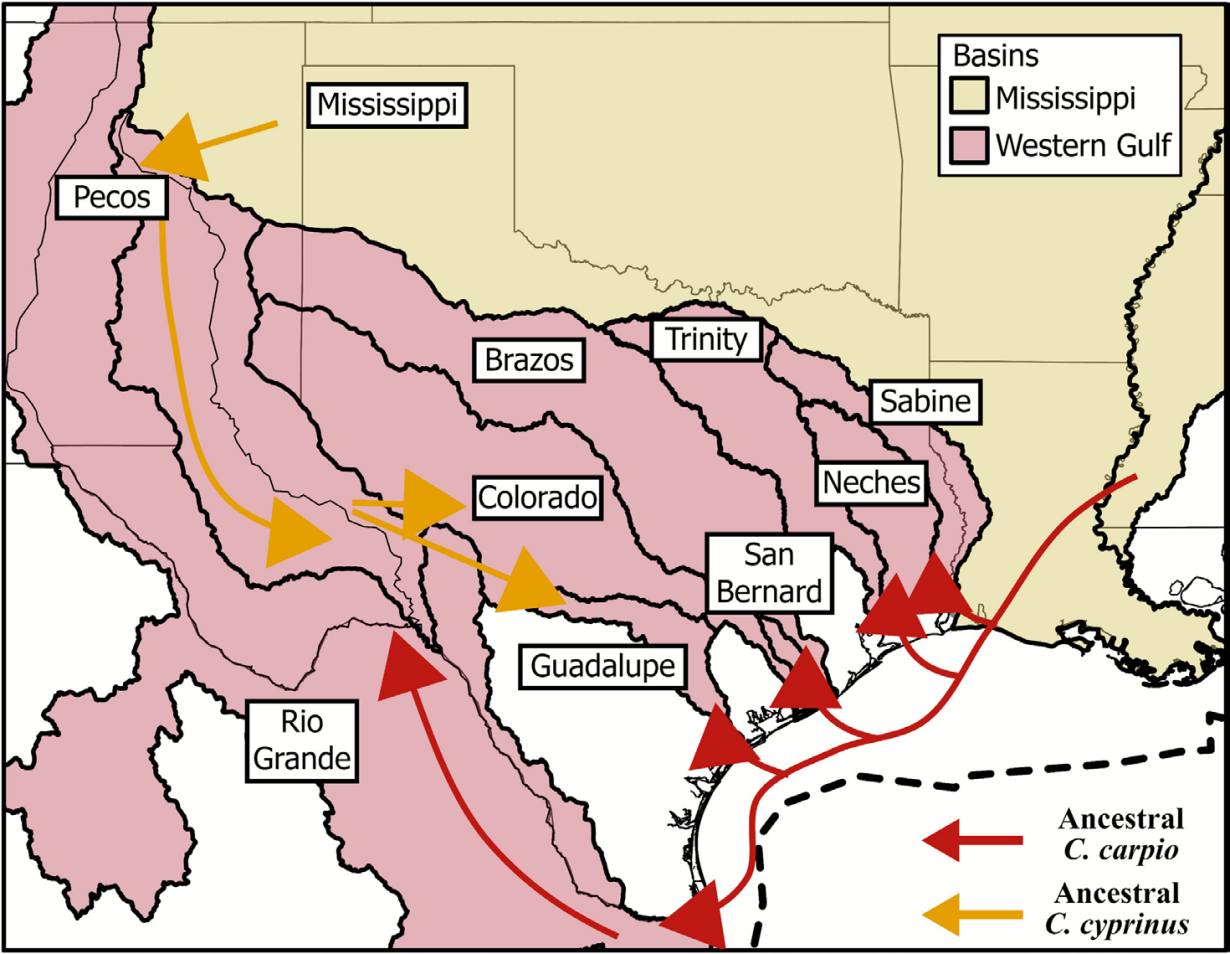
Conceptual diagram illustrating ancestral *Carpiodes* hypothesized biogeographic patterns leading to colonization of the Western Gulf of Mexico (i.e., Western Gulf) basin. Western Gulf drainages illustrated include the Brazos, Colorado, Guadalupe, Neches, Rio Grande, Sabine, San Bernard, and Trinity. The Pecos drainage is also illustrated nested within the Rio Grande drainage. Hypothesized dispersal routes for ancestral *Carpiodes* are shown. Ancestral *C. cyprinus* is hypothesized to colonize Western Gulf drainages originally when an ancient Red River tributary of the Mississippi basin was captured by the Middle Pecos River during the Pliocene and its tributaries were captured by Guadalupe and Colorado drainages (Schönhuth et al. 2012; MacGuigan et al. 2021; Hoagstrom and Echelle 2022). This was followed by the Pecos fully integrating with the Rio Grande in the Pleistocene (see Hoagstrom et al. 2025), leading to multiple colonization routes for ancestral *C. cyprinus* into Colorado, Guadalupe, and Rio Grande drainages. Ancestral *C. cyprinus* may have further colonized the San Bernard River (not shown) when it was originally an Early Holocene tributary to the Colorado River (Blum and Aslan 2006). Ancestral *C. carpio* may have colonized Western Gulf drainages following the Last Glacial Maximum when lower sea levels (thick dashed line) and increased glacial runoff allowed for expansion of Mississippi basin fishes west to the Rio Grande (Hoagstrom et al. 2025).

While Chen et al. (2010) suggest Colorado‐Rio Grande *Carpiodes* may be a unique species, we provide an alternative perspective. During the Pliocene, a tributary to the ancient Red River of the Mississippi basin was captured by the Middle Pecos River (Hoagstrom and Echelle 2022). Following this, Pliocene Pecos tributaries were captured by Colorado and Guadalupe drainages, and the Pleistocene Pecos fully integrated with the Rio Grande (Schönhuth et al. 2012; Kim and Conway 2014; MacGuigan et al. 2021; Hoagstrom et al. 2025). These processes would have allowed ancestral *C. cyprinus* to colonize throughout Colorado, Guadalupe, and Rio Grande drainages (Figure 5), along with the San Bernard River when it was tributary to the Colorado in the Early Holocene (Blum and Aslan 2006). Isolation following river capture from Mississippi and Rio Grande drainages may have promoted speciation of ancestral *C. cyprinus* into morphologically similar LRCS, as seen in *Dionda flavipinnis* inhabiting Colorado‐Guadalupe drainages (Schönhuth et al. 2012). Alternatively, isolation may have led to the formation of regionally specific lineages between Mississippi and Western Gulf *C. cyprinus* , as in *Micropterus punctulatus* (Kim, Taylor, and Near 2022). Ancestral *C. carpio* may have occupied Western Gulf drainages during deglaciation of the Last Glacial Maximum (~20,000 years ago) when lower sea levels and increased glacial runoff allowed for expansion of Mississippi basin fishes west to the Rio Grande (French 2021; Hoagstrom et al. 2025). Considering ancestral *Carpiodes* are hypothesized to have different routes of colonization, we suspect Mississippi and Western Gulf *C. carpio* relative to Mississippi *C. cyprinus* and LRCS will have different evolutionary trajectories (i.e., lineages). Given the recent hypothesized colonization of *C. carpio* to Western Gulf drainages, Rio Grande *C. carpio* are likely closely related to other *C. carpio* populations and do not represent a unique species.

**FIGURE 6:**
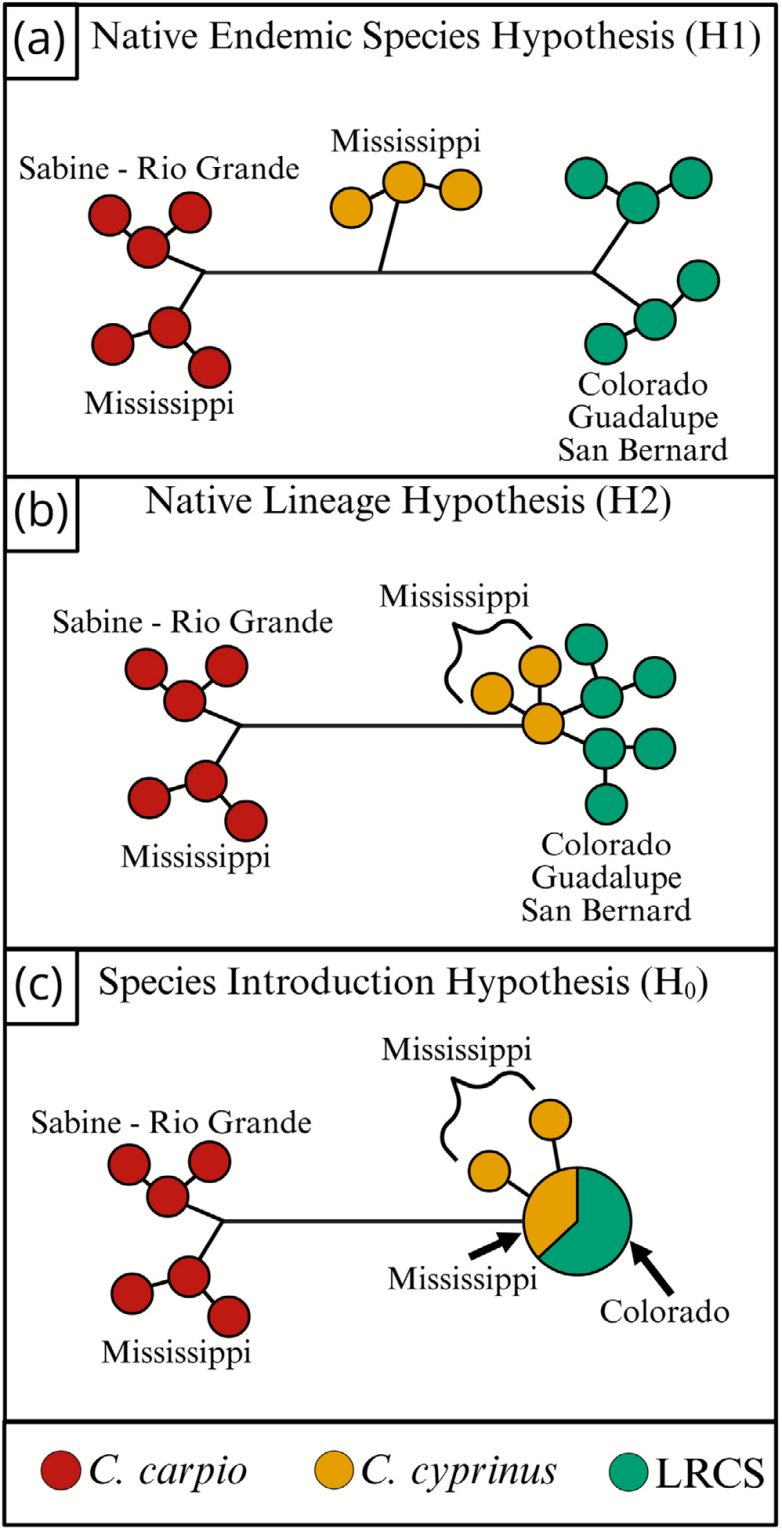
Competing molecular hypotheses assessing genetic patterns related to the identity of Llano River Carpsucker (LRCS). In each panel, we expect *Carpiodes carpio* of Mississippi and Western Gulf of Mexico (i.e., Western Gulf) basins to be more closely related to each other than Mississippi basin *Carpiodes cyprinus* or LRCS. Western Gulf drainages illustrated here include the Sabine west to the Rio Grande (Sabine‐Rio Grande). (a) Native Endemic Species Hypotheses (H1). Llano River Carpsucker are an undescribed species sharing more recent ancestry with Mississippi *C. cyprinus* than Mississippi and Western Gulf *C. carpio* but are distantly related to *C. carpio* and *C. cyprinus*. (b) Native Lineage Hypothesis (H2). Llano River Carpsucker are native *C. cyprinus* closely related to *C. cyprinus* from the Mississippi drainage and distantly related to Mississippi and Western Gulf *C. carpio*. (c) Species Introduction Hypothesis (H_0_). Llano River Carpsucker of the Colorado drainage are non‐native *C. cyprinus* that share a lineage with the source population of their introduction, such as from the Mississippi basin. Figure created with BioRender.com (https://biorender.com/).

With historical biogeographic scenarios in mind, it is possible to test competing molecular hypotheses to understand patterns of lineage diversification leading to LRCS. Our Native Endemic Species Hypothesis (H1) suggests LRCS is an undescribed species distantly related to Mississippi *C. cyprinus* and *C. carpio* populations, where LRCS lineages share more recent ancestry with *C. cyprinus* (Figure 6a). Alternatively, our Native Lineage Hypothesis (H2) posits LRCS are native *C. cyprinus* with lineages closely related to Mississippi *C. cyprinus* and distantly related to *C. carpio* (Figure 6b).

**FIGURE A1:**
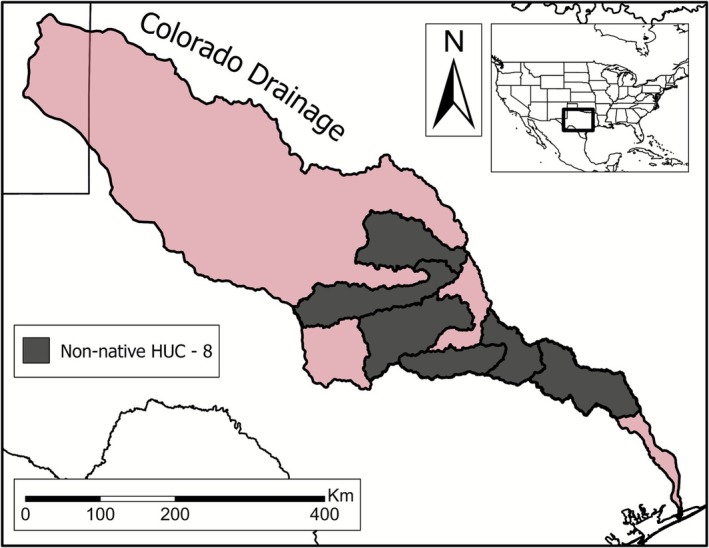
Non‐native occurrences of *Carpiodes cyprinus* in the Colorado drainage of Texas. Darker regions display watersheds at the Hydrologic Unit Code 8 (HUC‐8) level and denote where non‐native *C. cyprinus* occur according to provisional data housed at the U.S. Geological Survey Nonindigenous Aquatic Species database (Geological Survey 2025). Records indicate *C. cyprinus* may have been accidently stocked with sportfish in these HUC‐8 regions. Non‐native *C. cyprinus* in the Colorado drainage have been reported since 1963, with the original record corresponding with the the earliest collection of Llano River Carpsucker housed at the Fishes of Texas database (Hendrickson and Cohen 2022; U.S. Geological Survey 2025).

While the preceding hypotheses suggest LRCS are of native origin, the taxon may alternatively be non‐native *C. cyprinus* . According to provisional data supplied by the U.S. Geological Survey (USGS), *C. cyprinus* were stocked accidentally with sportfish throughout the Colorado drainage (Appendix: Figure A1). Considering *C. cyprinus* are known to invade novel habitats through incidental sportfish stocking (e.g., Hilling et al. 2018) these processes may also be happening in the Colorado drainage. If LRCS are non‐native *C. cyprinus* , LRCS of the Colorado drainage would share a lineage with *C. cyprinus* from their introductory source, such as the Mississippi basin (Figure 6c). However, no stocking records can substantiate claims of Western Gulf non‐native *C. cyprinus* , and this designation does not explain records of LRCS in Guadalupe and San Bernard drainages. Therefore, we consider this our null hypothesis which can be tested against our two competing alternative hypotheses suggesting the taxon is of native origin. It should be noted that our hypotheses do not account for hybridization across *Carpiodes* taxa, despite suckers readily hybridizing (e.g., Bart Jr. et al. 2010; Bangs et al. 2018). While hybridization patterns may complicate lineage relationships of *Carpiodes*, we believe our hypotheses still provide a solid framework for determining the identity of LRCS, and note any hybridization patterns while interpreting our data, should they occur.

The goal of our study was to determine the native and taxonomic status of LRCS using mitochondrial and nuclear markers. Additionally, we aimed to provide a preliminary hypothesis of phylogenetic intrarelationships for *Carpiodes*. Our objectives were to: (1) Test competing molecular hypotheses to determine the identity of LRCS for directing conservation and management of the taxon, and (2) Analyze preliminary *Carpiodes* phylogenetic relationships across the genus to provide further research directions for potential species descriptions. By assessing molecular hypotheses support, the classification and native status of LRCS can be informed and ultimately help understand evolutionary relationships across this taxonomically challenging group of fishes.

## 2 Methods

### 2.1 Tissue Acquisition

**FIGURE A2:**
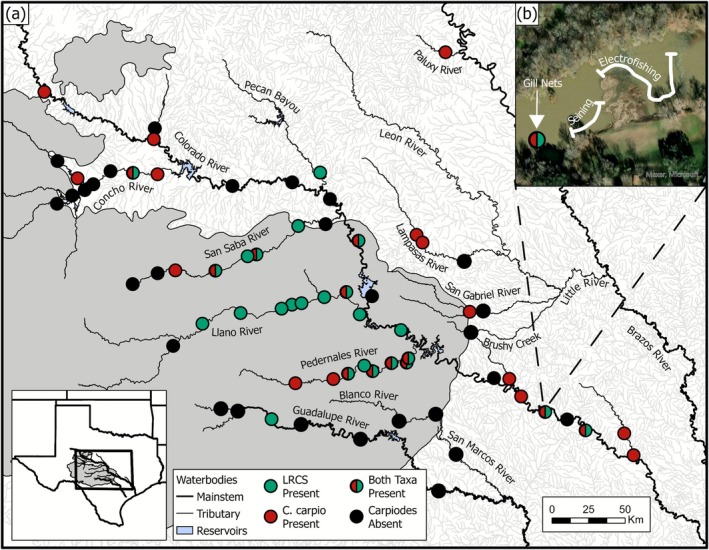
Distribution of field collections from the Brazos, Colorado, and Guadalupe drainages. (a) Colored circles denote locations where Llano River Carpsucker (LRCS) were collected, *Carpiodes carpio* were collected, or both taxa were collected. Black circles are locations where neither taxa were collected. The highlighted region in (a) denotes the extent of the Edwards Plateau ecoregion. (b) Illustration of the sampling strategy at each site using a combination of gill nets, seining, and electrofishing.

Tissues used for this study were acquired through a combination of field sampling and museum requests. During the summers of 2022 and 2023, *Carpiodes* were collected using seining, electrofishing, and gill nets at sites predominantly within the Edwards Plateau ecoregion of Texas. This semi‐arid ecoregion is known for its karst geology and clear, high‐gradient streams (Craig et al. 2016). We sampled sites across Colorado, Brazos, and Guadalupe drainages with a focused effort on tributaries of the Colorado drainage such as the Llano River (Appendix: Figure A2). Upon collection, a small piece of tissue comprising a segment of gill filament or paired‐fin tissue on smaller specimens (< 200 mm total length) was removed from the right side of the body and preserved in 100% laboratory‐grade ethanol (see *Further Reading‐1* for more information on field collection and specimen preservation techniques). Preserved specimens were identified as either *C. carpio* or LRCS using differences in mouth morphology and meristics. Specimens exhibiting intermediate characters were classified as LRCS. Field‐collected tissues were compiled with those obtained from museums across North America (see *Acknowledgments*).

### 2.2 Taxonomic Assignment

It was necessary to standardize the use of scientific names before analyzing our data. To avoid taxonomic confusion, we standardized all “cf.” taxa to their original scientific name (e.g., *Carpiodes* cf. *velifer* to *Carpiodes velifer* ) and reclassified subspecies. Several GenBank sequences (Sayers et al. 2021) originating from Rio Grande specimens classified as *C. c. elongatus* were classified as *C. carpio* . Additionally, two Rio Grande specimens from the University of Alabama Ichthyological Collection (UAIC 50866.01) determined as *C. cyprinus* were classified as *C. carpio* . Inspection of these specimens revealed they had weakly developed lower lip protuberances indicative of Rio Grande *C. carpio* . Additionally, sequences deposited to GenBank classified as *Carpiodes* cf. *cyprinus* from the Colorado drainage were referred to as LRCS. These sequences stem from specimens collected on the Llano River, the same system LRCS were originally collected, and are deposited at the Royal D. Suttkus Fish Collection of Tulane University (TU 198401, TU 198406). Reclassified names for all specimens used in this study are summarized in Table S1.

### 2.3 Laboratory Techniques

Deoxy‐ribonucleic acid (DNA) extraction from compiled tissues was conducted using procedures from the Omega Bio‐tek E.Z.N.A. DNA Kit. Extractions were used to amplify the entire 1140 base pair (bp) mitochondrial cytochrome *b* gene (CYTB) and first exon of the 849 bp nuclear interphotoreceptor retinoid‐binding protein gene 2 (IRBP2). While CYTB is traditionally used, we elected to use the nuclear IRBP2 gene as it is known to resolve phylogenetic relationships of suckers (Chen and Mayden 2012). Polymerase chain reaction (PCR) amplicons for each gene included a mixture of 1× buffer (3.75 μL), 2 mM MgCl_2_ (2.25 μL), 0.2 mM of each dNTP (0.4308 μL), 10 mM primer (0.405 μL), Taq Polymerase (0.1875 μL), DNA free water (10.3217 μL), and 1–3 μL of DNA depending on tissue quality. For CYTB, primers included forward GLU (5´‐TAACCGAGACCAATGACTTG‐3′) and reverse THR (5´‐ATCTTCGGATTACAAGACCG‐3′) oligonucleotides following Clements et al. (2012). Amplification thermal cycles included an initial denaturation stage at 94°C for 1 min; 35 cycles of denaturation at 94°C for 30 s, annealing at 57°C for 30 s, extension at 72°C for 30 s; followed by a final extension stage at 72°C for 10 min. For IRBP2, primers included forward IRBP2 101F (5´–TCMTGGACAAYTACTGCTCACC–3′) and reverse IRBP2 1068R (5´–AGATCAKGYTGTATTCCCCACTA–3′) oligonucleotides designed by Chen et al. (2008). Amplification followed a thermal cycle provided by Hunt et al. (2021) including an initial denaturation at 95°C for 2 min; 45 cycles of denaturation at 95°C for 1 min, annealing at 55°C for 1 min, extension at 72°C for 2 min; followed by a final extension stage at 72°C for 10 min. Successful PCR amplification was verified using gel electrophoresis and a DNA ladder. Prior to sequencing, 0.9 μL of ExoSAP‐IT PCR Product Cleanup Reagent was added to PCR amplicons to remove smaller DNA fragments. Sanger sequencing of amplicons was performed by Psomagen USA (Rockville, MD).

### 2.4 Sequence Processing

Contiguous sequences from corresponding forward and reverse chromatograms were de novo assembled using Geneious Prime (v2023.2.1). Manual editing of contiguous sequences was implemented to assess erroneous base pairs along with gaps and insertions produced by the nucleotide assembly. Contiguous sequences were transformed into consensus sequences and then aligned using the Multiple Sequence Comparison by Log‐ Expectation (MUSCLE) algorithm implemented in Geneious Prime. Amino acid translations of each sequence were assessed to confirm a lack of premature stop codons and aid in trimming our sequences to reference *Carpiodes* sequences (1140 bp for CYTB; 839 bp for IRBP2) gathered from GenBank. Our sequences were then MUSCLE aligned with GenBank sequences originating from specimens housed in the Royal D. Suttkus Fish Collection of Tulane University (CYTB = 90; IRBP2 = 2). Nuclear IRBP2 sequences were further processed to account for heterozygous sites. Using DNA Sequence Polymorphism (v6.12.03) software, IRBP2 sequences were phased into single haplotypes (i.e., IRBP2 A and IRBP2 B). GenBank accession numbers and associated metadata from all specimens in this study can be found in Table S1.

### 2.5 Statistical Analysis

#### 2.5.1 Phylogenetics

Phylogenetic methods were performed to determine the relationships of *Carpiodes*. Ideally, all specimens included in our study would have both nuclear and mitochondrial sequences. However, tissues associated with GenBank sequences from Tulane University were destroyed (H. L. Bart, *Personal Communication*) in 2005 when Hurricane Katrina struck New Orleans and were unavailable for additional IRBP2 sequence development.

Extraction failure led to additional sequence discrepancies but was rare. Typically, these discrepancies (*N* = 10) arose when a given tissues CYTB product sequenced successfully but their IRBP2 product did not (see Table S2 for further clarity). Considering discrepancies in sample size, we fit a series of consensus trees to incorporate all combinations of available data. Initially, we fit a single gene CYTB tree which included all available specimens with CYTB sequences (*N* = 425), referred to as the full tree. Following this, we fit additional trees using a subset of specimens from the full tree that had both CYTB and IRBP2 sequences (*N* = 327), referred to as subsetted trees. Using these sequences, we fit another single gene CYTB tree, an IRBP2 tree, and a tree concatenating both CYTB and IRBP2 genes (i.e., Concatenated tree). Considering that Concatenated and IRBP2 trees utilized phased nuclear sequences, it was necessary to determine whether specimens were heterozygous or homozygous across IRBP2. Heterozygous specimens were represented with two sequences (see Zhang et al. 2019), while homozygous individuals were represented with one sequence. Therefore, Concatenated tree sequences utilized both CYTB + IRBP2 A and CYTB + IRBP2 B sequences when specimens were heterozygous, or just CYTB + IRBP2 A sequences when specimens were homozygous. The subsetted IRBP2 tree followed the same pattern as above but did not incorporate CYTB sequences.

To develop consensus trees, models of nucleotide substitution used for phylogenetic analyses were determined using jModelTest2 v2.1.6 (Darriba et al. 2012) implemented with the CyberInfrastructure for Phylogenetic RESearch (CIPRES) Science Gateway portal (v3.3; Miller et al. 2010). Using jModelTest2, we selected default parameters and inferred the best‐supported model using Akaike Information Criterion (AIC; Akaike 1974). Top models comprised GTR + I + G, GTR + G, TVMef + I + G, and TIM3 + I + G for full CYTB and subsetted CYTB, IRBP2, and Concatenated trees, respectively (Table 2). Model descriptions and assumptions include: (1) Transversion model—equal base frequencies (TVMef), variable transversion rates, equal transition rates, equal base frequencies; (2) Transition model (TIM3), variable transition rates, two transversion rates, unequal base frequencies; and (3) General Time Reversible (GTR), variable transition and transversion rates, unequal base frequencies. Additional Invariable (+I) and Gamma (+G) parameters reflect models assuming invariable nucleotide sites, and variation in nucleotide evolution among sites, respectively.

**TABLE 2:** Summary statistics for the four consensus phylogenetic trees developed during this study. The first tree is composed of all specimens with mitochondrial cytochrome *b* (CYTB) sequences (i.e., Full). Remaining trees are composed of a subset of specimens where CYTB and nuclear interphotoreceptor retinoid‐binding protein gene 2 (IRBP2) sequences were available (i.e., Subsetted). While the number of specimens was equally represented across subsetted trees, sequences used in IRBP2 and Concatenated trees were higher than the subsetted CYTB tree, as they incorporated specimens with heterozygous IRBP2 sequences that were represented twice. For each tree, the number of sequences, number of specimens, number of characters (Length), variable sites, parsimony informative characters (Parsimony Sites), and best fitting model of nucleotide evolution determined through Akaike Information Criterion (AIC) are reported. TVMef and TIM3 models were substituted with GTR for phylogenetic inference. Further reading on models of nucleotide substitution can be found in Darriba and Posada (2014).

	Full	Subsetted		
	CYTB	CYTB	IRBP2	Concatenated
Number of sequences	425	327	481	481
Number of specimens	425	327	327	327
Length	1140	1140	839	1979
Variable sites	250	203	50	253
Parsimony sites	176	115	26	151
Best fitting model (AIC)	GTR + I + G	GTR + G	TVMef+I + G	TIM3 + I + G

Phylogenetic inference was developed using MrBayes v3.2.7a (Ronquist et al. 2012) in CIPRES. TVMef and TIM based models were substituted with GTR for phylogenetic analysis as they are unavailable in MrBayes and nested within the parameter rich GTR model, which infrequently creates differences in topology (Hoff et al. 2016). Selected nucleotide substitution models were used to parameterize Bayesian implemented trees, which used two independent Markov Chain Monte Carlo (MCMC) simulations each with 50,000,000 generations, a 10% burn‐in period, and a tree sampling interval of 1000. Parameters of sampled trees were checked for stationarity using Tracer v1.7.2 (Rambaut et al. 2018). We considered parameters stationary if their effective sample size exceeded 200 (Drummond et al. 2006). Following this, we used the “sumt” function in MrBayes to summarize sampled trees, where each posterior probability (PP) at an internal node of the consensus tree demonstrates the proportion of sampled trees with the same given node. Consensus trees were visualized using FigTree (v1.4.4) and Boxy SVG (v4.55.0). Each tree was outgroup rooted with a single *Ictiobus bubalus* specimen sequenced for CYTB and IRBP2.

#### 2.5.2 Genetic Distance Estimation

Genetic distances were estimated to assess levels of divergence between *Carpiodes*. Estimates were initially calculated by determining average genetic distances across all major clades. However, preliminary analysis of the full CYTB tree demonstrated the majority (97%) of specimens were resolved as members of a single large clade (i.e., Clade 3). Therefore, we performed additional genetic distance analyses across subclades of Clade 3. Tests included raw proportion of nucleotide substitution differences (*p*‐distance), and Nei's standard genetic distances (Nei's *D*). All *p*‐distance analyses were conducted in MEGA (v11.0.13). Calculation of Nei's *D* was conducted using the ‘genet.dist’ function of the *hierfstat* package in R studio (Goudet and Jombart 2022; R Core Team 2024).

#### 2.5.3 Haplotype Networks

Median‐joining haplotype networks were additionally developed from *Carpiodes* sequences using PopART v1.7 (Bandelt et al. 1999). For this analysis, we were interested in assessing hypothesized ancestral *Carpiodes* colonization routes to determine how these processes may relate to the genetics of LRCS. As biogeographic patterns indicate *C. carpio* and *C. cyprinus* colonized Western Gulf drainages from the Mississippi basin, we visualized haplotypes for all *C. carpio* , *C. cyprinus* , and LRCS specimens from Mississippi and Western Gulf basins with CYTB and IRBP2 sequences. For this analysis, all IRBP2 phased sequences were used, regardless of whether a specimen was homozygous or heterozygous (e.g., Eytan et al. 2009). To understand finer relationships across Mississippi and Western Gulf *Carpiodes*, we visualized *Carpiodes* haplotype relationships by categorizing sequences by taxonomic assignment and populations based on the drainage in which they were collected.

## 3 Results

### 3.1 Phylogenetic Analysis

Nine hundred six unique sequences (CYTB = 425; IRBP2 = 481) were used to assess phylogenetic relationships of *Carpiodes*. Sample sizes of specimens and associated sequences for each consensus tree are summarized in Table 2. Of the four consensus trees developed, all parameters calculated under combined MCMC chains exceeded an effective sample size of 200 after a 10% burn‐in period. Our full CYTB tree had the highest amount of parsimony informative sites. The lack of variable sites from the IRBP2 tree led to a single polytomy and was uninformative. Considering this, our interpretation of *Carpiodes* phylogenetic relationships relied on the full CYTB tree. Compared to this tree, subsetted CYTB and concatenated trees did not reveal major differences in topology (Figure S1; *Further Reading‐2*).

**FIGURE 7:**
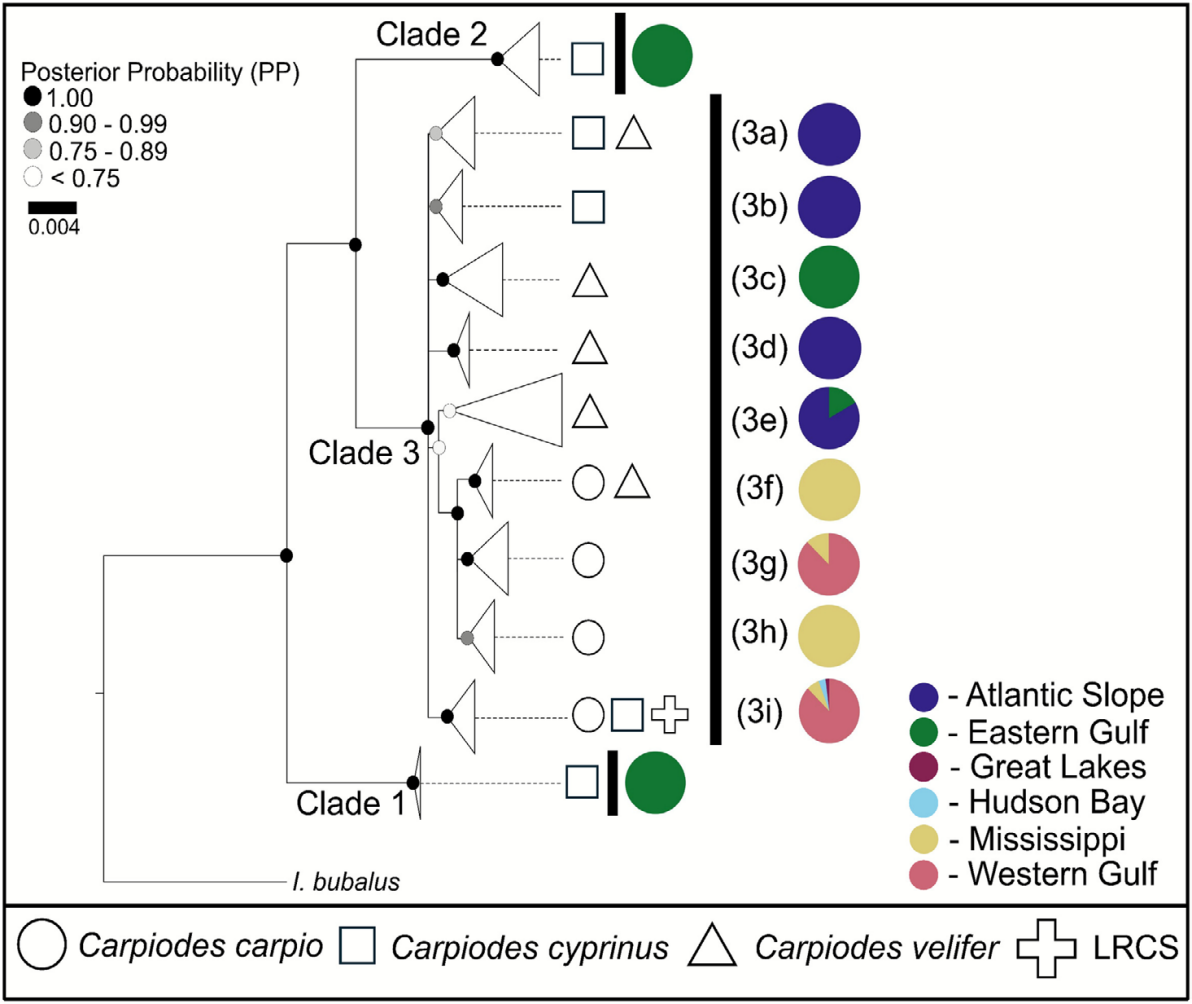
Collapsed Bayesian consensus tree assessing *Carpiodes* relationships of all specimens with mitochondrial cytochrome *b* (CYTB) sequences (i.e., full CYTB tree). The tree was outgroup rooted on an *Ictiobus bubalus* specimen from the Mississippi basin. At each internal node, the posterior probability (PP) is illustrated based on a range of values summarized above. Exact PP values for all internal nodes are reported in Figure S1. Major clades are labeled 1–3 with subclades in Clade 3 labeled 3a–3i. Pie charts denote the proportion of basins resolved in each major clade or subclade. Symbols next to major clades and subclades denote recognized *Carpiodes* taxa and Llano River Carpsucker (LRCS).

**FIGURE 8:**
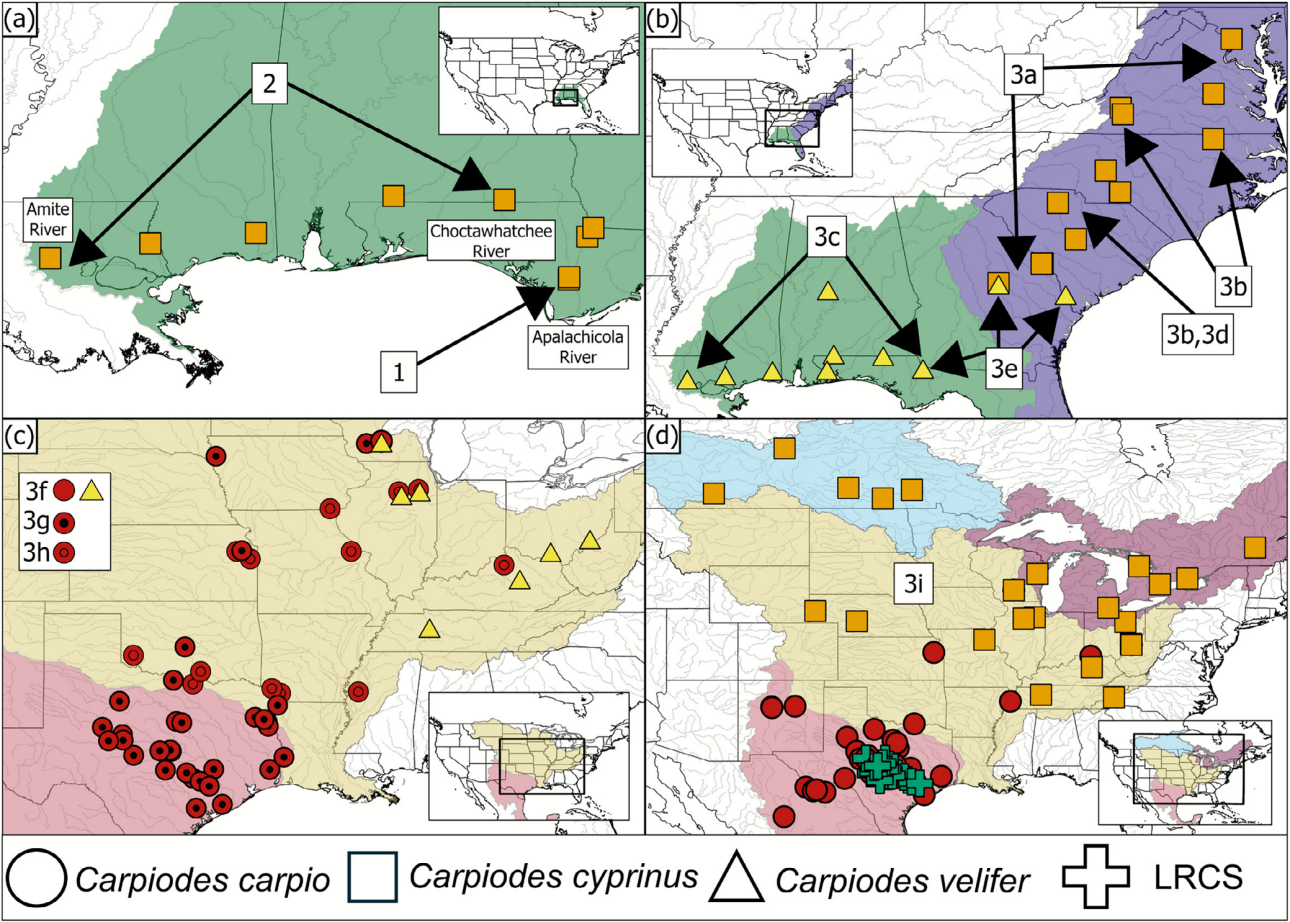
Distribution of *Carpiodes* major clades and subclades. Specimens are shape coded based on taxonomic assignment. (a) Distribution of *Carpiodes cyprinus* in the Eastern Gulf basin. Arrows broadly define the extent of Clades 1 and 2 in the Apalachicola River (Clade 1) and across Choctawhatchee‐Amite rivers (Clade 2). (b) Distribution of *C. cyprinus* and *Carpiodes velifer* in Atlantic Slope and Eastern Gulf basins. Arrows broadly define the extent of Subclades 3a–3e. (c) Distribution of *Carpiodes carpio* and *C. velifer* inhabiting Mississippi and Western Gulf basins. Solid shapes denote specimens resolved in Subclade 3f, circles with black dots display *C. carpio* from Subclade 3g, and circles with colored dots illustrate *C. carpio* from Subclade 3h. (d) Distribution of specimens resolved in Subclade 3i across Great Lakes, Hudson Bay, Mississippi, and Western Gulf basins. All Llano River Carpsucker (LRCS) were resolved in Subclade 3i.

Our full CYTB tree yielded three major clades. An expanded version of the tree presented here is illustrated in Figure S2. Excluding Clades 1 and 2, a polytomy united remaining lineages rendering phylogenetic relationships largely uncertain (Figure 7). There was unambiguous support (PP = 1) for the sister placement of Clade 1 to all remaining *Carpiodes* lineages. Clade 1 consisted of *C. cyprinus* from the Apalachicola River of the Eastern Gulf basin (Figure 8a). Excluding Clade 1, there was also unambiguous support for the sister relationship of Clade 2 to remaining *Carpiodes* lineages. Clade 2 consisted of *C. cyprinus* inhabiting the Eastern Gulf basin from the Choctawhatchee River west to the Amite River (Figure 8a).

Remaining *Carpiodes* lineages under Clade 3 demonstrated a combination of *Carpiodes* taxa across all basins assessed in this study. Subclades 3a–3e possessed a mixture of *C. cyprinus* and *C. velifer* inhabiting Atlantic Slope and Eastern Gulf basins (Figure 8b). Subclades 3f–3h included all *C. velifer* from the Mississippi basin and *C. carpio* from Mississippi and Western Gulf basins (Figure 8c). Two subclades included *C. carpio* unique to the Mississippi basin (3f, 3h) while an additional subclade included a mixture of Mississippi and Western Gulf basin *C. carpio* (3g). Subclade 3i was composed of all LRCS, *C. carpio* from Mississippi and Western Gulf basins, and *C. cyprinus* from Great Lakes, Hudson Bay, and Mississippi basins (Figure 8d). Within Subclade 3i, all LRCS were resolved in distinct lineages shared only with sympatric *C. carpio* (Figure S3). Additionally, Rio Grande *C. carpio* were observed sharing the same lineage as *C. cyprinus* from the Mississippi basin as they possessed identical CYTB sequences. The positions of these clades render all recognized species of *Carpiodes* paraphyletic.

### 3.2 Genetic Distance Estimation

Genetic distances were highest when contrasting Clade 1. Contrasts between Clades 1 and 2 resulted in a *p*‐distance = 0.071 (Nei's *D* = 0.219) while Clades 1 and 3 possessed a *p*‐distance = 0.059 (Nei's *D* = 0.172). Contrasts between Clades 2 and 3 resulted in a *p*‐distance = 0.055 (Nei's *D* = 0.154). Remaining genetic distances comparing subclades of Clade 3 (Table 3) were lower (*p*‐distance < 0.018; Nei's *D* < 0.045) except for those contrasting Subclade 3e (*p*‐distance = 0.028–0.033; Nei's *D* = 0.070–0.089). Lowest genetic distances comparing the subclade containing LRCS (i.e., Subclade 3i) included a contrast with Subclade 3b composed of *C. cyprinus* from the Atlantic Slope basin (*p*‐distance = 0.011; Nei's *D* = 0.016).

**TABLE 3:** Summary of mean Nei's standard genetic distances (Nei's *D*), below diagonal, and mean raw proportion of nucleotide differences (*p*‐distance), above diagonal, across subclades of *Carpiodes* resolved in Clade 3. Nine subclades were used for pairwise comparisons (Subclades 3a–3i). Each element of the matrix demonstrates the average Nei's *D* or *p*‐distance between two subclades.

	3a	3b	3c	3d	3e	3f	3g	3h	3i
3a	—	0.009	0.015	0.013	0.03	0.015	0.016	0.014	0.013
3b	0.016	—	0.012	0.012	0.028	0.012	0.013	0.011	0.011
3c	0.026	0.021	—	0.017	0.029	0.016	0.016	0.015	0.015
3d	0.026	0.027	0.035	—	0.033	0.017	0.016	0.016	0.014
3e	0.078	0.075	0.07	0.089	—	0.03	0.031	0.029	0.029
3f	0.034	0.03	0.033	0.043	0.083	—	0.008	0.006	0.013
3g	0.034	0.029	0.031	0.039	0.081	0.015	—	0.007	0.014
3h	0.03	0.025	0.028	0.039	0.078	0.012	0.011	—	0.012
3i	0.026	0.026	0.03	0.033	0.079	0.033	0.033	0.029	—

### 3.3 Haplotype Networks

**FIGURE 9:**
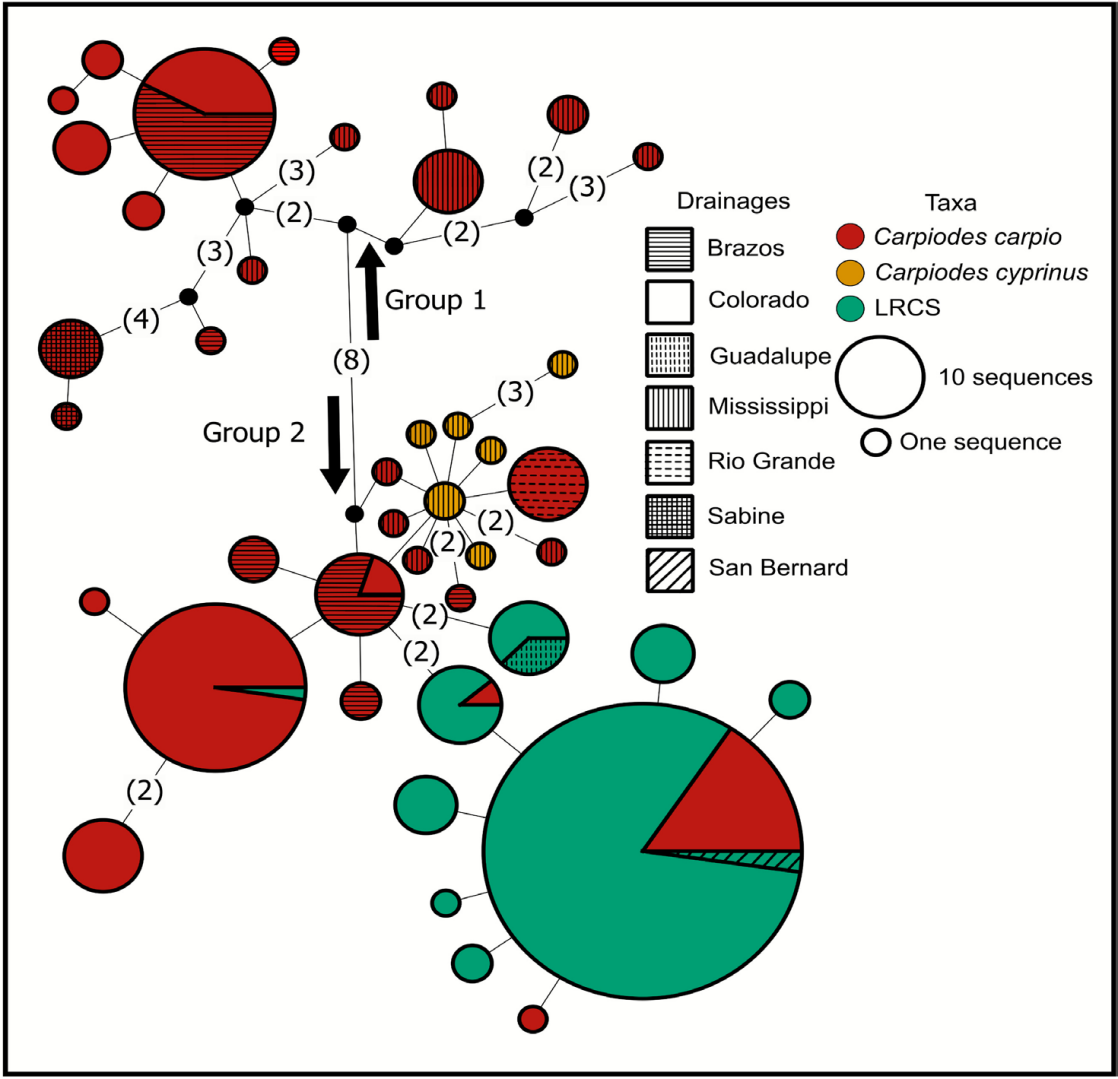
Median‐joining haplotype network of the mitochondrial cytochrome *b* (CYTB) gene investigating genetic relationships of *Carpiodes* from Mississippi and Western Gulf of Mexico (i.e., Western Gulf) basins. Each circle represents a unique haplotype with size proportional to the number of identical sequences. Colored haplotypes denote each taxa while different patterns illustrate which drainages specimens were collected in. Two major haplotype groups (i.e., Group 1, Group 2) were identified from this analysis indicated with arrows. Excluding one step mutations, mutational changes between each haplotype are given in parentheses.

Three hundred eight CYTB sequences across an equal number of specimens were utilized for our median‐joining CYTB haplotype network, comprising 42 unique haplotypes. We observed two main haplotype groups separated by eight mutations for CYTB (Figure 9). Group 1 consisted of *C. carpio* from the Brazos, Colorado, Mississippi, and Sabine drainages. Group 2 consisted of all LRCS and Mississippi *C. cyprinus* along with *C. carpio* from Brazos, Colorado, Mississippi, and Rio Grande drainages.

Within Group 1, haplotypes were unique to each drainage except for a common haplotype shared between Brazos and Colorado *C. carpio* . Within Group 2, the most common LRCS haplotype was four mutations from the most common *C. cyprinus* haplotype (*p*‐distance = 0.0035). This haplotype included most LRCS specimens from the Colorado drainage, all LRCS from the San Benard drainage, and was shared with Colorado *C. carpio* . The LRCS common haplotype was five mutations from a haplotype comprised of LRCS from Colorado and Guadalupe drainages (*p*‐distance = 0.0044). Within Group 2, we also observed haplotypes shared between Brazos and Colorado *C. carpio* . Additionally, Rio Grande *C. carpio* all possessed the same haplotype and were one mutation from the most common *C. cyprinus* haplotype. It should be noted that Rio Grande *C. carpio* also shared CYTB haplotypes with Mississippi *C. cyprinus* . However, we were unable to further assess these *C. cyprinus* specimens here as they did not have IRBP2 sequences.

**FIGURE 10:**
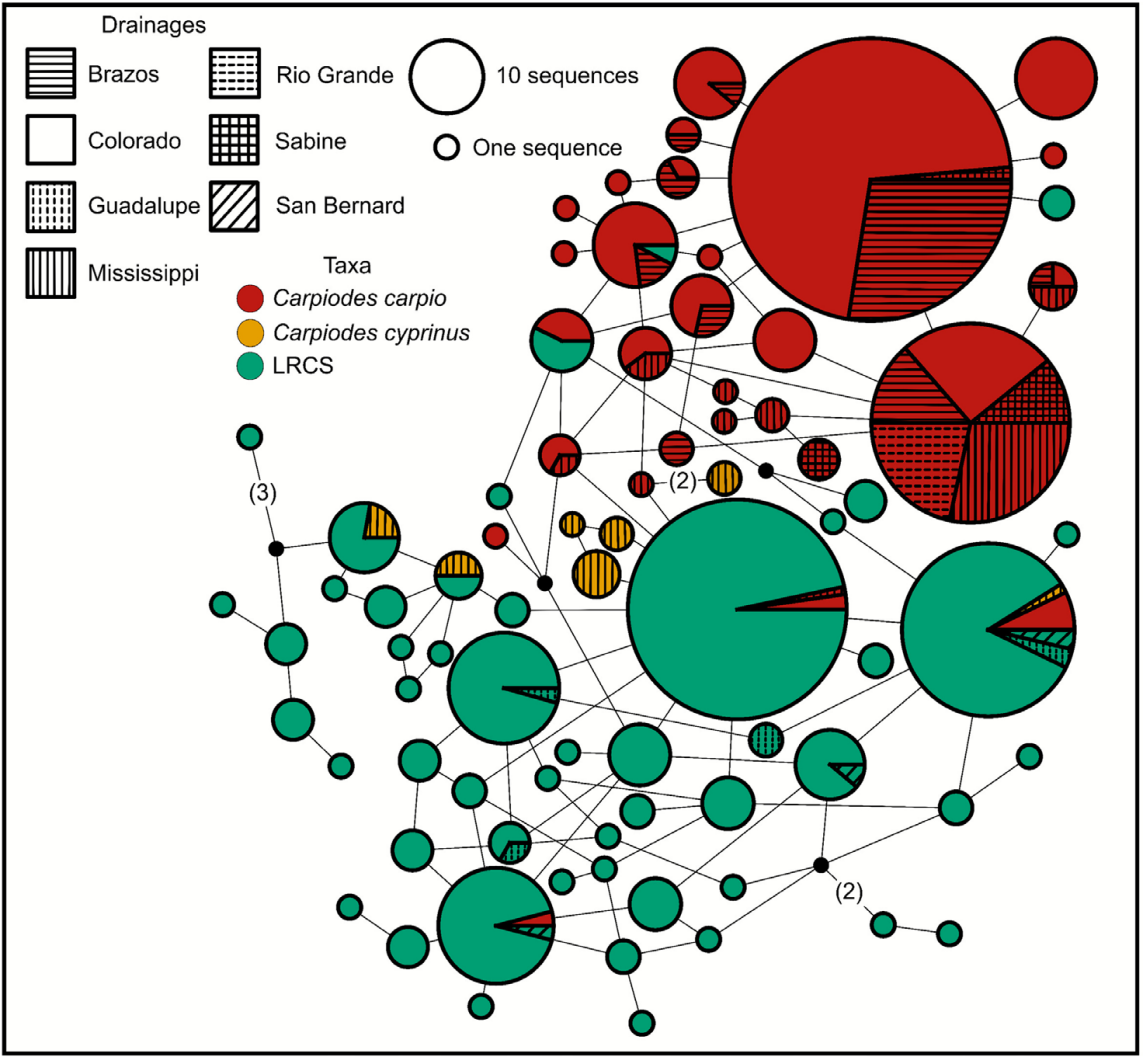
Median‐joining haplotype network from the nuclear interphotoreceptor retinoid‐binding protein gene 2 (IRBP2) gene investigating genetic relationships of *Carpiodes* from Mississippi and Western Gulf of Mexico (i.e., Western Gulf) basins. Each circle represents a unique haplotype with size proportional to the number of identical sequences. Colored haplotypes denote each taxa while different patterns illustrate which drainages specimens were collected in. Excluding one step mutations, mutational changes between each haplotype are given in parentheses.

Our IRBP2 haplotype dataset comprised 616 sequences represented across 308 specimens. Haplotype divergence of our IRBP2 dataset was low, with an average *p*‐distance = 0.004 across all individuals. Our IRBP2 haplotype network yielded 78 unique haplotypes. Despite low divergence, there was clearer separation between *C. carpio* haplotypes and those occupied by LRCS and *C. cyprinus* (Figure 10). A common *C. carpio* nuclear haplotype was shared across specimens inhabiting Brazos, Colorado, Mississippi, Rio Grande and Sabine drainages. The majority of IRBP2 haplotype diversity stemmed from private Colorado LRCS haplotypes. However, LRCS shared haplotypes with *C. cyprinus* from the Mississippi drainage, with LRCS from Guadalupe and San Bernard drainages, and with *C. carpio* from Brazos, Colorado, and Mississippi drainages.

## 4 Discussion

Our study investigated phylogenetic relationships across the geographic range of *Carpiodes* and assessed genetic patterns between Western Gulf and Mississippi populations with the goal of classifying LRCS and determining its native status. We provide evidence of early diverging clades of *C. cyprinus* in the Apalachicola and Choctawhatchee–Amite rivers of the Eastern Gulf basin suggesting *C. cyprinus* , as currently understood, is a cryptic species complex. These lineages correspond with other early diverging Eastern Gulf species such as *Moxosotoma lachneri* and *Micropterus henshalli* of Apalachicola and Mobile drainages (Bagley et al. 2018; Kim, Taylor, and Near 2022). Considering the long‐term geographical isolation of these drainages (see Hoagstrom et al. 2014), it is unsurprising that divergent *Carpiodes* occur here as well. Species descriptions for these taxa are essential, as conservation actions often fail to protect undescribed species (Ely et al. 2017).

Our work further provides evidence behind the identity of LRCS. Specimens possessed unique CYTB lineages relative to all *C. cyprinus* populations with CYTB haplotypes being minimally divergent from Mississippi *C. cyprinus* . We interpret this evidence as support for our Native Lineage Hypotheses (H2). That is, LRCS are native *C. cyprinus*, with our genetic patterns displaying conspecific population structure. This finding is similar to Langille et al. (2016) where genetic analysis of *Catostomus catostomus* displayed minimally divergent CYTB haplotypes (*p*‐distance ≤ 0.01) across vast geographic distances (i.e., British Columbia to Labrador, Canada). However, our haplotype networks suggest additional genetic processes occur between *Carpiodes* taxa warranting further investigation, along with genetic mechanisms behind Rio Grande *C. carpio* .

Although Mississippi and Western Gulf *C. cyprinus* (formerly LRCS) mitochondrial haplotypes were distinct, their nuclear haplotypes were shared. While identical haplotypes across separate populations may point to species introductions as in Glotzbecker et al. (2016) and support our Species Introduction Hypothesis (H_0_), a more likely explanation is incomplete lineage sorting. Incomplete lineage sorting is commonly used to explain shared haplotypes in other cypriniform fishes (Tang et al. 2012; Dvořák et al. 2022). In our study, we demonstrate Mississippi and Western Gulf *C. cyprinus* lineages are sorted for CYTB, but IRBP2 haplotypes still retain shared ancestral polymorphisms. These findings are similar to Corona‐Santiago et al. (2018), where investigation of the sucker genus *Pantosteus* revealed incomplete lineage sorting for the nuclear growth hormone copy I gene, but lineages were sorted for CYTB.

Our study also observed shared CYTB haplotypes between Colorado *C. carpio* and sympatric *C. cyprinus* despite taxa typically possessing separate nuclear IRBP2 haplotypes. This finding suggests the mitochondrial genome of Colorado *C. carpio* has been replaced with that of *C. cyprinus* via mitochondrial introgression. Mitochondrial introgression is widely documented across the diversity of life for many vertebrate groups (McGuire et al. 2007; Keck and Near 2010; Seixas et al. 2018). Additionally, fishes that experience mitochondrial introgression often do not have reciprocal changes in nuclear loci, such as in salmonids (e.g., Wilson and Bernatchez 1998), and the majority of *C. carpio* in this study.

Brazos, Colorado, and Mississippi *C. carpio* were also observed to have genetically distant or similar CYTB haplotypes relative to *C. cyprinus* . We interpret this pattern as parental (i.e., genetically distant) and additionally introgressed lineages of *C. carpio* occurring across these drainages, indicative of hybrid regions according to Harrison and Larson (2014). However, considering mitochondrial introgression posits the replacement of mitochondrial DNA by a donor species, additional shared haplotypes between introgressed and parental specimens should be present. In the Brazos, a lack of this pattern may be due to historical mitochondrial introgression between *C. carpio* and extinct lineages of *C. cyprinus* . Bernatchez et al. (1995) suggested Canadian populations of *Salvelinus fontinalis* experienced mitochondrial fixation by currently allopatric *Salvelinus alpinus* due to historical mitochondrial introgression when populations were sympatric. We suspect similar patterns led to genetically similar Brazos *C. carpio* relative to *C. cyprinus* if the taxon was historically present in this drainage. These processes may also explain genetically similar Colorado *C. carpio* haplotypes relative to sympatric *C. cyprinus* , as populations may have historically introgressed with extinct *C. cyprinus* lineages. Alternatively in the Mississippi, we suspect lower sample sizes may explain why CYTB haplotypes were not shared between currently sympatric *C. carpio* and *C. cyprinus* as additional parental *C. cyprinus* lineages likely exist but were not sampled. These findings expand literature on the porous nature of species boundaries (e.g., Harrison and Larson 2014; Barraclough 2024) and suggest both parental and introgressed lineages of *C. carpio* can cooccur across much of their distribution.

Rio Grande *C. carpio* shared CYTB haplotypes with *C. cyprinus* of the Mississippi but retained separate nuclear haplotypes relative to *C. cyprinus* . These observations suggest Rio Grande *C. carpio* are genetically *C. cyprinus* for mitochondrial DNA but are *C. carpio* for nuclear DNA, possibly due to historical mitochondrial introgression and incomplete lineage sorting. Pliocene capture of an ancient Red River tributary by the Pecos River and Pleistocene integration of the Pecos with the Rio Grande likely facilitated hybridization between ancestral *Carpiodes* when ancestral *C. carpio* reached the Rio Grande following the Last Glacial Maximum (Hoagstrom and Echelle 2022; Hoagstrom et al. 2025). Considering deglaciation of the Last Glacial Maximum was geologically recent (~20,000 years ago; French 2021), mitochondrial introgression of Rio Grande *C. carpio* with ancestral *C. cyprinus* must also be. This explains why introgressed Rio Grande *C. carpio* share CYTB haplotypes with Mississippi *C. cyprinus* , as not enough time has passed to completely sort Mississippi and Rio Grande populations. Interestingly, Rio Grande *C. carpio* with their weakly developed or absent lower lip protuberance and 35–37 lateral‐line scales demonstrate morphological characteristics approaching *C. cyprinus* . We hypothesize these characteristics may be due to hybridization leading to a breakdown of species barriers between ancestral *C. carpio* and *C. cyprinus* , as documented in other groups of fishes (e.g., Taylor et al. 2006). This may also explain the apparent absence of *C. cyprinus* in the Rio Grande, as the two parental taxa may have merged into a hybrid population possessing intermediate morphological characteristics. However, further sampling in the Rio Grande would be necessary to confirm this along with genetic techniques that allow for analysis of multiple nuclear loci.

Patterns of hypothesized ancestral routes of *Carpiodes* colonization to Western Gulf drainages are further supported by findings in this study. Ancestral *C. cyprinus* were expected to originally colonize Western Gulf drainages when an ancient Red River tributary of the Mississippi basin was captured by the Pliocene Pecos and its tributaries were captured by Colorado–Guadalupe drainages (Schönhuth et al. 2012; MacGuigan et al. 2021; Hoagstrom et al. 2025). These patterns explain why Mississippi and Western Gulf *C. cyprinus* are closely related, as they share recent ancestry and have exhibited lineage diversification following isolation. Regarding *C. carpio* , ancestral *C. carpio* were hypothesized to colonize Western Gulf drainages following the Last Glacial Maximum when lower sea levels and increased glacial runoff allowed for expansion of Mississippi basin fishes west to the Rio Grande (Hoagstrom et al. 2025). These processes likely would have facilitated recent introgression (~20,000 years ago) in regions of secondary contact (e.g., Colorado, Brazos, Rio Grande) between ancestral *C. carpio* and *C. cyprinus* , an idea supported by hybrid *C. carpio* occurring in these drainages. Additionally, these colonization routes explain the absence of *C. cyprinus* in certain Western Gulf drainages such as the Sabine, where *C. carpio* occur and display no evidence of hybridization with *C. cyprinus* .

While our study has important taxonomic implications, the genetic patterns we discovered can also direct future conservation and management of Texas *Carpiodes*. According to USGS provisional data, *C. cyprinus* is considered non‐native in Texas. However, we provide evidence of native Western Gulf *C. cyprinus* . This finding fundamentally changes the view that *C. cyprinus* in Texas might represent another example of the growing concern over unmeasured effects of non‐native transplant suckers (Hartman and Larson 2023). Instead, the limited and disjunct distribution of *C. cyprinus* in Texas represents populations on the peripheral range for the species. Such peripheral populations are critical features of evolution given their propensity to be genetically or morphologically unique and are often afforded conservation measures (Lesica and Allendorf 1995). Under this perspective, biologists in Texas may consider promoting *C. cyprinus* to a Species of Greatest Conservation Need, which could ultimately lead to state protection for better management of populations.

Several caveats and limitations need to be discussed for this study. Much of our genetic inferences rely on a single mitochondrial gene. Single or multi‐locus studies are now commonly replaced with next generation sequencing (NGS) techniques (e.g., Harris et al. 2005; Kim, Bauer, and Near 2022). Future studies incorporating NGS techniques may advance understanding of phylogenetic intrarelationships of this genus as currently they are unclear. Furthermore, NGS studies may provide a stronger understanding of introgression patterns compared to our findings, as sufficiently more nuclear loci will allow for more complex analyses of hybridization patterns (e.g., population admixture analysis). Additionally, our lack of a resolved phylogenetic hypothesis prevented us from conducting divergence times analyses to further test biogeographic hypotheses relating to the patterns observed between Mississippi and Western Gulf *Carpiodes*. Lastly, it is unclear what drivers may be causing selection of slender Western Gulf *C. cyprinus* . The majority of Western Gulf *C. cyprinus* were collected in the Edwards Plateau ecoregion of Texas, a geologically unique region with high gradient streams that may be causing selection of their slender form. Bailey and Allum (1962) suggested that variation in *C. cyprinus* morphology may be linked to environmental variables, but this remains untested. Future studies should incorporate morphometrics and investigate how they relate to environmental variables, such as those conducted in Foster et al. (2015) to determine possible sources of selection on body shape.

## 5 Conclusion

Our study presents important genetic and taxonomic information for the conservation of the genus *Carpiodes*. Additionally, through multiple lines of evidence we recognize LRCS as native *C. cyprinus* and provide recommendations to inform management and conservation of Western Gulf populations. In a world that is increasingly susceptible to biodiversity loss, taxonomic studies are not only essential for describing new species, but also for refining the geographic range of known species. This in turn can proliferate conservation and management of native fishes, which ultimately helps maintain biodiversity. Our study of *Carpiodes* populations serves as an example of how resolving cryptic diversity may further our understanding of biogeographic and evolutionary patterns of North American freshwater fishes.

## Author Contributions

**H. C. Roberts:** conceptualization (equal), data curation (lead), formal analysis (lead), investigation (equal), methodology (equal), visualization (lead), writing – original draft (lead), writing – review and editing (lead). **P. T. Bean:** conceptualization (equal), formal analysis (supporting), funding acquisition (equal), investigation (equal), writing – review and editing (equal). **K. D. Keith:** conceptualization (supporting), formal analysis (supporting), investigation (equal), writing – review and editing (equal). **K. W. Conway:** conceptualization (equal), formal analysis (equal), funding acquisition (equal), writing – review and editing (equal). **J. S. Perkin:** conceptualization (equal), data curation (equal), formal analysis (equal), funding acquisition (lead), investigation (equal), methodology (equal), visualization (equal), writing – review and editing (equal).

## Conflicts of Interest

All authors declare no competing interests.

## Supporting information


**Data S1:** ece372543‐sup‐0001‐supinfo.zip.

## Data Availability

All of the data associated with this publication can be found on DRYAD: https://doi.org/10.5061/dryad.mw6m9069d.
