# Supplementary material for: The Carpiodes Conundrum: Molecular Hypothesis Testing Informs Conservation Applications for Carpsuckers (Catostomidae: Carpiodes) in Texas and Beyond
Source: Ecol Evol. 2025 Nov 23;15(11):e72543. doi: 10.1002/ece3.72543 (PMC12640881; doi:10.1002/ece3.72543)
Supplement: Supplementary file 1 — Data S1: ece372543‐sup‐0001‐supinfo.zip. [file ECE3-15-e72543-s001.zip › Roberts_et_al_2025_Supplementary.docx]

**Title:** The *Carpiodes* Conundrum: Molecular Hypothesis Testing Informs Conservation Applications for Carpsuckers (Catostomidae: *Carpiodes*) in Texas and Beyond

**Authors and Affiliations:**

Roberts, H.C.^1*^, Bean, P.T.^2^, Keith, K.D.^1^, Conway, K.W.^1^, and Perkin_,_ J.S.^1^

^1^Department of Ecology and Conservation Biology, Texas A&M University, 2258 TAMU, College Station, TX 77843

^2^ Heart of the Hills Fisheries Science Center, Inland Fisheries Division, Texas Parks and Wildlife Department, 5103 Junction Hwy, Mountain Home, TX 78058

*Corresponding Author

**Supplementary Information for Roberts et al. (2025)**

# **Figures:**


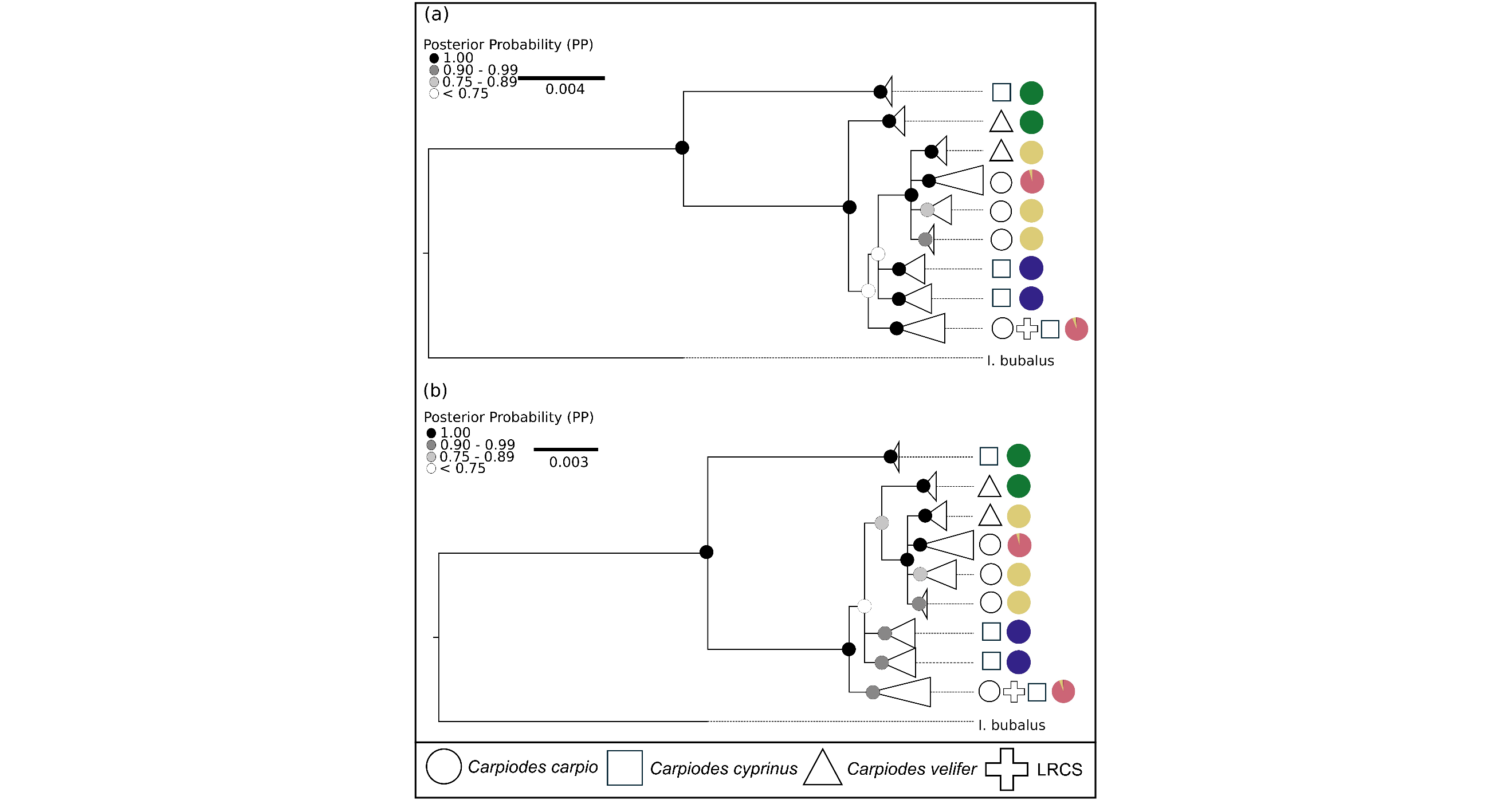


Figure S1. Collapsed Bayesian implemented phylogenetic trees showing relationships between *Carpiodes* lineages using a subset of specimens from the full tree (i.e., Figure 7 of main text). Collapsed lineages are color coded as in the main text tree. In each, internal nodes are color coded with a range of posterior probabilities (PP) illustrated. Large circles correspond to the proportion of basins represented under each clade. In (a) the subsetted cytochrome b (CYTB) tree is shown. In (b) the subsetted Concatenated tree is illustrated. Both trees utilize the same subset of specimens (N=327) where CYTB and nuclear interphotoreceptor retinoid-binding protein gene 2 (IRBP2) sequences were available. While the Concatenated tree used the same amount of specimens as (a), specimens with heterozygous IRBP2 alleles were represented twice (i.e. CYTB + IRBP2 A, CYTB + IRBP2 B) leading to a total of 481 sequences versus 327 sequences represented in (a). Interpretation of these phylogenies with respect to the main text tree can be found in *Further Reading – 2*.

Figure S2. See “Figure_S2.svg” file.

Figure S3. “Figure_S3.svg” file.

# **Tables:**

Table S1. Metadata of specimens and associated sequences. For each specimen, the taxon name is given followed by the sequence identifier (ID) and specimen voucher. For sequences that were reclassified, the original taxon ID is given, followed by the ID used in this study. Following this, GenBank accession numbers are provided for mitochondrial cytochrome b (CYTB) and nuclear interphotoreceptor retinoid-binding protein gene 2 (IRBP2) sequences. Locality information is also provided, including the principal drainage and Global Positioning System coordinates from where each specimen was collected.

| Taxon | ID | Specimen Voucher | Original ID | Reclassified ID | CYTB | IRBP2 | Drainage | Latitude | Longitude |
| --- | --- | --- | --- | --- | --- | --- | --- | --- | --- |
| **Genus *Ictiobus*** |  |  |  |  |  |  |  |  |  |
| *I. bubalus* | TU 124.07 | - | - | - | FJ226285 | JX488940 | Mississippi | - | - |
| **Genus *Carpiodes*** |  |  |  |  |  |  |  |  |  |
| *C. carpio* | 0350F | TCWC 20802.01 | - | - | PV579408 | PV579668 | Brazos | 31.07606 | -98.01089 |
| *C. carpio* | 0339F | TCWC 20801.01 | - | - | PV579409 | PV579669 | Brazos | 31.12428 | -98.05227 |
| *C. carpio* | 0342F | TCWC 20802.01 | - | - | PV579410 | PV579670 | Brazos | 31.07606 | -98.01089 |
| *C. carpio* | 0343F | TCWC 20802.01 | - | - | PV579411 | PV579671 | Brazos | 31.07606 | -98.01089 |
| *C. carpio* | 0344F | TCWC 20802.01 | - | - | PV579412 | PV579672 | Brazos | 31.07606 | -98.01089 |
| *C. carpio* | 0345F | TCWC 20802.01 | - | - | PV579413 | PV579673 | Brazos | 31.07606 | -98.01089 |
| *C. carpio* | 0346F | TCWC 20802.01 | - | - | PV579414 | PV579674 | Brazos | 31.07606 | -98.01089 |
| *C. carpio* | 0347F | TCWC 20802.01 | - | - | PV579415 | PV579675 | Brazos | 31.07606 | -98.01089 |
| *C. carpio* | 0349F | TCWC 20802.01 | - | - | PV579416 | PV579676 | Brazos | 31.07606 | -98.01089 |
| *C. carpio* | 0340F | TCWC 20802.01 | - | - | PV579417 | PV579677 | Brazos | 31.07606 | -98.01089 |
| *C. carpio* | 0348F | TCWC 20802.01 | - | - | PV579418 | PV579678 | Brazos | 31.07606 | -98.01089 |
| *C. carpio* | 0182F | TCWC 20803.01 | - | - | PV579419 | PV579679 | Brazos | 32.24889 | -97.81823 |
| *C. carpio* | 0184F | TCWC 20803.01 | - | - | PV579420 | PV579680 | Brazos | 32.24889 | -97.81823 |
| *C. carpio* | 0187F | TCWC 20803.01 | - | - | PV579421 | PV579681 | Brazos | 32.24889 | -97.81823 |
| *C. carpio* | 0193F | TCWC 20803.01 | - | - | PV579422 | PV579682 | Brazos | 32.24889 | -97.81823 |
| *C. carpio* | 0194F | TCWC 20803.01 | - | - | PV579423 | PV579683 | Brazos | 32.24889 | -97.81823 |
| *C. carpio* | 0183F | TCWC 20803.01 | - | - | PV579424 | PV579684 | Brazos | 32.24889 | -97.81823 |
| *C. carpio* | 0192F | TCWC 20803.01 | - | - | PV579425 | PV579685 | Brazos | 32.24889 | -97.81823 |
| *C. carpio* | 0177F | TCWC 20803.01 | - | - | PV579426 | PV579686 | Brazos | 32.24889 | -97.81823 |
| *C. carpio* | 0185F | TCWC 20803.01 | - | - | PV579427 | PV579687 | Brazos | 32.24889 | -97.81823 |
| *C. carpio* | 0189F | TCWC 20803.01 | - | - | PV579428 | PV579688 | Brazos | 32.24889 | -97.81823 |
| *C. carpio* | 0196F | TCWC 20804.01 | - | - | PV579429 | PV579689 | Brazos | 30.64315 | -97.68236 |
| *C. carpio* | 0195F | TCWC 20804.01 | - | - | PV579430 | PV579690 | Brazos | 30.64315 | -97.68236 |
| *C. carpio* | TCWC_19677.01 | TCWC 19677.01 | - | - | PV579741 | PV579807 | Brazos | 30.36823 | -96.34011 |
| *C. carpio* | TCWC_20259.02 | TCWC 20259.02 | - | - | PV579743 | PV579809 | Brazos | 32.2 | -97.6 |
| *C. carpio* | TCWC_20350.06 | TCWC 20350.06 | - | - | PV579744 | PV579810 | Brazos | 33.56 | -99.51 |
| *C. carpio* | TCWC_20352.03B | TCWC 20352.03 | - | - | PV579745 | PV579811 | Brazos | 33.01 | -101.4 |
| *C. carpio* | TNHC_467 | TNHC 52101 | - | - | PV579749 | PV579815 | Brazos | 32.79769 | -98.18677 |
| *C. carpio* | TNHC_479 | TNHC 51842 | - | - | PV579750 | PV579816 | Brazos | 32.61544 | -97.92551 |
| *C. carpio* | TNHC_565 | TNHC 55166 | - | - | PV579751 | PV579817 | Brazos | 33.04779 | -100.06124 |
| *C. carpio* | TNHC_1945 | TNHC 65719 | - | - | PV579753 | PV579819 | Brazos | 30.83326 | -96.948495 |
| *C. carpio* | TU 222.01 | TU 198403 | - | - | JN053239 | - | Colorado | 29.696494 | -96.516734 |
| *C. carpio* | TU 242.03 | TU 198405 | - | - | JN053245 | - | Colorado | 30.49226 | -99.75655 |
| *C. carpio* | TU 245.01 | TU 198411 | - | - | JN053248 | - | Colorado | 29.01674 | -96.00961 |
| *C. carpio* | TU 245.02 | TU 198411 | - | - | JN053249 | - | Colorado | 29.01674 | -96.00961 |
| *C. carpio* | TU 245.03 | TU 198411 | - | - | JN053250 | - | Colorado | 29.01674 | -96.00961 |
| *C. carpio* | TU 245.04 | TU 198411 | - | - | JN053251 | - | Colorado | 29.01674 | -96.00961 |
| *C. carpio* | 0257Y | TCWC 20601.01 | - | - | PV579190 | PV579450 | Colorado | 30.77785 | -98.5625 |
| *C. carpio* | 0261Y | TCWC 20601.01 | - | - | PV579191 | PV579451 | Colorado | 30.77785 | -98.5625 |
| *C. carpio* | 0266Y | TCWC 20601.01 | - | - | PV579192 | PV579452 | Colorado | 30.77785 | -98.5625 |
| *C. carpio* | 0270Y | TCWC 20601.01 | - | - | PV579193 | PV579453 | Colorado | 30.77785 | -98.5625 |
| *C. carpio* | 0175Y | TCWC 20607.01 | - | - | PV579244 | PV579504 | Colorado | 30.91917 | -99.78716 |
| *C. carpio* | 0174Y | TCWC 20608.01 | - | - | PV579245 | PV579505 | Colorado | 30.91668 | -99.49983 |
| *C. carpio* | 0167Y | TCWC 20610.01 | - | - | PV579251 | PV579511 | Colorado | 31.01509 | -99.20449 |
| *C. carpio* | 0616F | TCWC 20612.01 | - | - | PV579254 | PV579514 | Colorado | 32.01974 | -100.73622 |
| *C. carpio* | 0617F | TCWC 20612.01 | - | - | PV579255 | PV579515 | Colorado | 32.01974 | -100.73622 |
| *C. carpio* | 0619F | TCWC 20612.01 | - | - | PV579256 | PV579516 | Colorado | 32.01974 | -100.73622 |
| *C. carpio* | 0621F | TCWC 20612.01 | - | - | PV579257 | PV579517 | Colorado | 32.01974 | -100.73622 |
| *C. carpio* | 0615F | TCWC 20612.01 | - | - | PV579258 | PV579518 | Colorado | 32.01974 | -100.73622 |
| *C. carpio* | 0618F | TCWC 20612.01 | - | - | PV579259 | PV579519 | Colorado | 32.01974 | -100.73622 |
| *C. carpio* | 0620F | TCWC 20612.01 | - | - | PV579260 | PV579520 | Colorado | 32.01974 | -100.73622 |
| *C. carpio* | 0622F | TCWC 20612.01 | - | - | PV579261 | PV579521 | Colorado | 32.01974 | -100.73622 |
| *C. carpio* | 0623F | TCWC 20612.01 | - | - | PV579262 | PV579522 | Colorado | 32.01974 | -100.73622 |
| *C. carpio* | 0624F | TCWC 20612.01 | - | - | PV579263 | PV579523 | Colorado | 32.01974 | -100.73622 |
| *C. carpio* | 0611F | TCWC 20613.01 | - | - | PV579264 | PV579524 | Colorado | 31.72981 | -99.94179 |
| *C. carpio* | 0614F | TCWC 20613.01 | - | - | PV579265 | PV579525 | Colorado | 31.72981 | -99.94179 |
| *C. carpio* | 0606F | TCWC 20613.01 | - | - | PV579266 | PV579526 | Colorado | 31.72981 | -99.94179 |
| *C. carpio* | 0609F | TCWC 20613.01 | - | - | PV579267 | PV579527 | Colorado | 31.72981 | -99.94179 |
| *C. carpio* | 0602F | TCWC 20613.01 | - | - | PV579268 | PV579528 | Colorado | 31.72981 | -99.94179 |
| *C. carpio* | 0605F | TCWC 20613.01 | - | - | PV579269 | PV579529 | Colorado | 31.72981 | -99.94179 |
| *C. carpio* | 0607F | TCWC 20613.01 | - | - | PV579270 | PV579530 | Colorado | 31.72981 | -99.94179 |
| *C. carpio* | 0610F | TCWC 20613.01 | - | - | PV579271 | PV579531 | Colorado | 31.72981 | -99.94179 |
| *C. carpio* | 0613F | TCWC 20613.01 | - | - | PV579272 | PV579532 | Colorado | 31.72981 | -99.94179 |
| *C. carpio* | 0603F | TCWC 20613.01 | - | - | PV579273 | PV579533 | Colorado | 31.72981 | -99.94179 |
| *C. carpio* | 0759F | TCWC 20614.01 | - | - | PV579274 | PV579534 | Colorado | 31.0938 | -98.4705 |
| *C. carpio* | 0756F | TCWC 20614.01 | - | - | PV579275 | PV579535 | Colorado | 31.0938 | -98.4705 |
| *C. carpio* | 0760F | TCWC 20614.01 | - | - | PV579276 | PV579536 | Colorado | 31.0938 | -98.4705 |
| *C. carpio* | 0761F | TCWC 20615.01 | - | - | PV579279 | PV579539 | Colorado | 31.51328 | -99.91557 |
| *C. carpio* | 0763F | TCWC 20615.01 | - | - | PV579280 | PV579540 | Colorado | 31.51328 | -99.91557 |
| *C. carpio* | 0764F | TCWC 20615.01 | - | - | PV579281 | PV579541 | Colorado | 31.51328 | -99.91557 |
| *C. carpio* | 0765F | TCWC 20616.01 | - | - | PV579282 | PV579542 | Colorado | 31.52156 | -100.09285 |
| *C. carpio* | 0769F | TCWC 20616.01 | - | - | PV579283 | PV579543 | Colorado | 31.52156 | -100.09285 |
| *C. carpio* | 0767F | TCWC 20616.01 | - | - | PV579284 | PV579544 | Colorado | 31.52156 | -100.09285 |
| *C. carpio* | 0770F | TCWC 20616.01 | - | - | PV579285 | PV579545 | Colorado | 31.52156 | -100.09285 |
| *C. carpio* | 0774F | TCWC 20617.01 | - | - | PV579287 | PV579547 | Colorado | 31.48712 | -100.49291 |
| *C. carpio* | 0775F | TCWC 20617.01 | - | - | PV579288 | PV579548 | Colorado | 31.48712 | -100.49291 |
| *C. carpio* | 0826F | TCWC 20617.01 | - | - | PV579289 | PV579549 | Colorado | 31.48712 | -100.49291 |
| *C. carpio* | 0835F | TCWC 20617.01 | - | - | PV579290 | PV579550 | Colorado | 31.48712 | -100.49291 |
| *C. carpio* | 0840F | TCWC 20617.01 | - | - | PV579291 | PV579551 | Colorado | 31.48712 | -100.49291 |
| *C. carpio* | 0771F | TCWC 20617.01 | - | - | PV579292 | PV579552 | Colorado | 31.48712 | -100.49291 |
| *C. carpio* | 0772F | TCWC 20617.01 | - | - | PV579293 | PV579553 | Colorado | 31.48712 | -100.49291 |
| *C. carpio* | 0773F | TCWC 20617.01 | - | - | PV579294 | PV579554 | Colorado | 31.48712 | -100.49291 |
| *C. carpio* | 0827F | TCWC 20617.01 | - | - | PV579295 | PV579555 | Colorado | 31.48712 | -100.49291 |
| *C. carpio* | 0828F | TCWC 20617.01 | - | - | PV579296 | PV579556 | Colorado | 31.48712 | -100.49291 |
| *C. carpio* | 0829F | TCWC 20617.01 | - | - | PV579297 | PV579557 | Colorado | 31.48712 | -100.49291 |
| *C. carpio* | 0830F | TCWC 20617.01 | - | - | PV579298 | PV579558 | Colorado | 31.48712 | -100.49291 |
| *C. carpio* | 0831F | TCWC 20617.01 | - | - | PV579299 | PV579559 | Colorado | 31.48712 | -100.49291 |
| *C. carpio* | 0832F | TCWC 20617.01 | - | - | PV579300 | PV579560 | Colorado | 31.48712 | -100.49291 |
| *C. carpio* | 0833F | TCWC 20617.01 | - | - | PV579301 | PV579561 | Colorado | 31.48712 | -100.49291 |
| *C. carpio* | 0834F | TCWC 20617.01 | - | - | PV579302 | PV579562 | Colorado | 31.48712 | -100.49291 |
| *C. carpio* | 0837F | TCWC 20617.01 | - | - | PV579303 | PV579563 | Colorado | 31.48712 | -100.49291 |
| *C. carpio* | 0838F | TCWC 20617.01 | - | - | PV579304 | PV579564 | Colorado | 31.48712 | -100.49291 |
| *C. carpio* | 0843F | TCWC 20617.01 | - | - | PV579305 | PV579565 | Colorado | 31.48712 | -100.49291 |
| *C. carpio* | 0844F | TCWC 20617.01 | - | - | PV579306 | PV579566 | Colorado | 31.48712 | -100.49291 |
| *C. carpio* | 0845F | TCWC 20617.01 | - | - | PV579307 | PV579567 | Colorado | 31.48712 | -100.49291 |
| *C. carpio* | 0847F | TCWC 20617.01 | - | - | PV579308 | PV579568 | Colorado | 31.48712 | -100.49291 |
| *C. carpio* | 0849F | TCWC 20618.01 | - | - | PV579309 | PV579569 | Colorado | 30.24113 | -98.66364 |
| *C. carpio* | 0021F | TCWC 20619.01 | - | - | PV579310 | PV579570 | Colorado | 30.21804 | -98.93408 |
| *C. carpio* | 0023F | TCWC 20619.01 | - | - | PV579311 | PV579571 | Colorado | 30.21804 | -98.93408 |
| *C. carpio* | 0025F | TCWC 20619.01 | - | - | PV579312 | PV579572 | Colorado | 30.21804 | -98.93408 |
| *C. carpio* | 0005F | TCWC 20620.01 | - | - | PV579313 | PV579573 | Colorado | 30.27266 | -98.5582 |
| *C. carpio* | 0009F | TCWC 20620.01 | - | - | PV579314 | PV579574 | Colorado | 30.27266 | -98.5582 |
| *C. carpio* | 0034F | TCWC 20622.01 | - | - | PV579336 | PV579596 | Colorado | 30.28636 | -98.38299 |
| *C. carpio* | 0043F | TCWC 20622.01 | - | - | PV579337 | PV579597 | Colorado | 30.28636 | -98.38299 |
| *C. carpio* | 0048F | TCWC 20622.01 | - | - | PV579338 | PV579598 | Colorado | 30.28636 | -98.38299 |
| *C. carpio* | 0630F | TCWC 20622.01 | - | - | PV579339 | PV579599 | Colorado | 30.28636 | -98.38299 |
| *C. carpio* | 0029F | TCWC 20623.01 | - | - | PV579348 | PV579608 | Colorado | 30.3342 | -98.24779 |
| *C. carpio* | 0801F | TCWC 20623.01 | - | - | PV579349 | PV579609 | Colorado | 30.3342 | -98.24779 |
| *C. carpio* | 0814F | TCWC 20624.01 | - | - | PV579355 | PV579615 | Colorado | 30.3358 | -98.13906 |
| *C. carpio* | 0818F | TCWC 20624.01 | - | - | PV579356 | PV579616 | Colorado | 30.3358 | -98.13906 |
| *C. carpio* | 0817F | TCWC 20624.01 | - | - | PV579357 | PV579617 | Colorado | 30.3358 | -98.13906 |
| *C. carpio* | 0820F | TCWC 20625.01 | - | - | PV579360 | PV579620 | Colorado | 30.36237 | -98.12634 |
| *C. carpio* | 0821F | TCWC 20625.01 | - | - | PV579361 | PV579621 | Colorado | 30.36237 | -98.12634 |
| *C. carpio* | 0091F | TCWC 20627.01 | - | - | PV579374 | PV579634 | Colorado | 30.22279 | -97.40994 |
| *C. carpio* | 0089F | TCWC 20628.01 | - | - | PV579375 | PV579635 | Colorado | 30.11293 | -97.32896 |
| *C. carpio* | 0090F | TCWC 20628.01 | - | - | PV579376 | PV579636 | Colorado | 30.11293 | -97.32896 |
| *C. carpio* | 0080F | TCWC 20629.01 | - | - | PV579377 | PV579637 | Colorado | 30.01293 | -97.16016 |
| *C. carpio* | 0083F | TCWC 20629.01 | - | - | PV579378 | PV579638 | Colorado | 30.01293 | -97.16016 |
| *C. carpio* | 0082F | TCWC 20629.01 | - | - | PV579379 | PV579639 | Colorado | 30.01293 | -97.16016 |
| *C. carpio* | 0078F | TCWC 20629.01 | - | - | PV579380 | PV579640 | Colorado | 30.01293 | -97.16016 |
| *C. carpio* | 0081F | TCWC 20629.01 | - | - | PV579381 | PV579641 | Colorado | 30.01293 | -97.16016 |
| *C. carpio* | 0237F | TCWC 20630.01 | - | - | PV579386 | PV579646 | Colorado | 29.89238 | -96.87729 |
| *C. carpio* | 0242F | TCWC 20630.01 | - | - | PV579387 | PV579647 | Colorado | 29.89238 | -96.87729 |
| *C. carpio* | 0076F | TCWC 20630.01 | - | - | PV579388 | PV579648 | Colorado | 29.89238 | -96.87729 |
| *C. carpio* | 0238F | TCWC 20630.01 | - | - | PV579389 | PV579649 | Colorado | 29.89238 | -96.87729 |
| *C. carpio* | 0239F | TCWC 20630.01 | - | - | PV579390 | PV579650 | Colorado | 29.89238 | -96.87729 |
| *C. carpio* | 0244F | TCWC 20630.01 | - | - | PV579391 | PV579651 | Colorado | 29.89238 | -96.87729 |
| *C. carpio* | 0235F | TCWC 20631.01 | - | - | PV579400 | PV579660 | Colorado | 29.73029 | -96.54147 |
| *C. carpio* | 0236F | TCWC 20631.01 | - | - | PV579401 | PV579661 | Colorado | 29.73029 | -96.54147 |
| *C. carpio* | 0162Y | TCWC 20632.01 | - | - | PV579402 | PV579662 | Colorado | 29.86719 | -96.60358 |
| *C. carpio* | 0163Y | TCWC 20632.01 | - | - | PV579403 | PV579663 | Colorado | 29.86719 | -96.60358 |
| *C. carpio* | 0164Y | TCWC 20632.01 | - | - | PV579404 | PV579664 | Colorado | 29.86719 | -96.60358 |
| *C. carpio* | 0165Y | TCWC 20632.01 | - | - | PV579405 | PV579665 | Colorado | 29.86719 | -96.60358 |
| *C. carpio* | TU 244.01 | TU 198409 | - | - | JN053246 | - | Guadalupe | 28.83204 | -97.03168 |
| *C. carpio* | TU 244.02 | TU 198409 | - | - | JN053247 | - | Guadalupe | 28.83204 | -97.03168 |
| *C. carpio* | TU 104.07 | - | - | - | JN053177 | - | Mississippi | 43.29361 | -89.72028 |
| *C. carpio* | TU 112.06 | TU 192315 | - | - | JN053185 | - | Mississippi | 38.416159 | -84.880582 |
| *C. carpio* | TU 116.09 | TU 194140 | - | - | JN053187 | - | Mississippi | 43.29361 | -89.72028 |
| *C. carpio* | TU 116.11 | TU 194140 | - | - | JN053188 | - | Mississippi | 43.29361 | -89.72028 |
| *C. carpio* | TU 117.05 | TU 192324 | - | - | JN053190 | - | Mississippi | 38.677922 | -84.328186 |
| *C. carpio* | TU 121.08 | TU 194157 | - | - | JN053193 | - | Mississippi | 43.205 | -90.315 |
| *C. carpio* | TU 123.07 | TU 196612 | - | - | JN053194 | - | Mississippi | 33.4187 | -90.63378 |
| *C. carpio* | TU 164.02 | - | - | - | JN053208 | - | Mississippi | - | - |
| *C. carpio* | TU 211.01 | TU 196732 | - | - | JN053222 | - | Mississippi | 32.89277 | -93.82091 |
| *C. carpio* | TU 219.01 | - | - | - | JN053237 | - | Mississippi | 33.72872 | -97.16207 |
| *C. carpio* | TU 220.10 | TU 198207 | - | - | JN053238 | - | Mississippi | 35.18787 | -97.4763 |
| *C. carpio* | TU 248.01 | - | - | - | JN053252 | - | Mississippi | 33.878164 | -97.933852 |
| *C. carpio* | TU 248.02 | - | - | - | JN053253 | - | Mississippi | 33.878164 | -97.933852 |
| *C. carpio* | TU 250.01 | - | - | - | JN053255 | - | Mississippi | 34.215893 | -96.856268 |
| *C. carpio* | TU 251.01 | - | - | - | JN053256 | - | Mississippi | 34.858272 | -99.508461 |
| *C. carpio* | FMNH_118313.01 | FMNH 118313 | - | - | PV579700 | PV579775 | Mississippi | 41.39 | -88.2575 |
| *C. carpio* | FMNH_118314 | FMNH 118314 | - | - | PV579701 | PV579776 | Mississippi | 41.3019 | -89.0375 |
| *C. carpio* | KUI_416 | KUI 32213 | - | - | PV579706 | PV579780 | Mississippi | 38.9761 | -95.2354 |
| *C. carpio* | KUI_2533 | KUI 22940 | - | - | PV579707 | PV579781 | Mississippi | 38.9784 | -95.2329 |
| *C. carpio* | KUI_7876 | KUI 39523 | - | - | PV579708 | PV579782 | Mississippi | 38.953476 | -95.431148 |
| *C. carpio* | KUI_8240 | KUI 39527 | - | - | PV579709 | PV579783 | Mississippi | 38.655328 | -94.89961 |
| *C. carpio* | KUI_8260 | KUI 39844 | - | - | PV579710 | PV579784 | Mississippi | 38.932026 | -95.330451 |
| *C. carpio* | KUI_8793 | Photo Voucher |  |  | PV579711 | PV579785 | Mississippi | 42.702466 | -96.251187 |
| *C. carpio* | NCSM_37355.1 | NCSM 37355 | - | - | PV579717 | PV579789 | Mississippi | 33.363 | -93.7016 |
| *C. carpio* | NCSM_47102.1 | NCSM 47102 | - | - | PV579721 | PV579792 | Mississippi | 35.5193 | -89.9648 |
| *C. carpio* | OSUM_116748 | OSUM 116748 | - | - | PV579729 | PV579800 | Mississippi | 39.4676 | -81.4864 |
| *C. carpio* | TCWC_20356.01 | TCWC 20356.01 | - | - | PV579746 | PV579812 | Mississippi | 33.88 | -97.93 |
| *C. carpio* | TNHC_440 | TNHC 49569 | - | - | PV579748 | PV579814 | Mississippi | 33.5513 | -94.04502 |
| *C. carpio* | TNHC_1226 | TNHC 62119 | - | - | PV579752 | PV579818 | Mississippi | 33.9417 | -96.7007 |
| *C. carpio* | UAIC_50868.01 | UAIC 50868.01 | - | - | PV579762 | PV579829 | Mississippi | 40.63814 | -91.74953 |
| *C. carpio* | UAIC_50869.01 | UAIC 50869.01 | - | - | PV579763 | PV579830 | Mississippi | 38.95162 | -90.91453 |
| *C. carpio* | TU 256.02 | TU 200821 | - | - | JN053259 | - | Neches | 30.35694 | -94.13017 |
| *C. carpio* | TU 142.01 | - | *C. c. elongatus* | *C. carpio* | JN053201 | - | Rio Grande | 27.46 | -105.82 |
| *C. carpio* | TU 212.01 | - | *C. c. elongatus* | *C. carpio* | JN053223 | - | Rio Grande | 35.13 | -106.69 |
| *C. carpio* | TU 249.03 | - | *C. c. elongatus* | *C. carpio* | JN053254 | - | Rio Grande | 29.520007 | -104.290835 |
| *C. carpio* | TU 254.01 | - | *C. c. elongatus* | *C. carpio* | JN053257 | - | Rio Grande | 35.072619 | -106.664113 |
| *C. carpio* | TCWC_16456.15 | TCWC 16456.15 | - | - | PV579735 | PV579804 | Rio Grande | 30.12811 | -101.5732 |
| *C. carpio* | TCWC_17178.01 | TCWC 17178.01 | - | - | PV579736 | - | Rio Grande | 29.17767 | -102.998 |
| *C. carpio* | TCWC_17182.01 | TCWC 17182.01 | - | - | PV579737 | - | Rio Grande | 29.31508 | -103.5512 |
| *C. carpio* | TCWC_17183.04 | TCWC 17183.04 | - | - | PV579738 | - | Rio Grande | 29.32712 | -103.5542 |
| *C. carpio* | TCWC_17557.08 | TCWC 17557.08 | - | - | PV579739 | PV579805 | Rio Grande | 29.31485 | -103.5514 |
| *C. carpio* | TCWC_17559.09 | TCWC 17559.09 | - | - | PV579740 | PV579806 | Rio Grande | 29.52 | -104.29 |
| *C. carpio* | TCWC_19726.07 | TCWC 19726.07 | - | - | PV579742 | PV579808 | Rio Grande | 29.25631 | -103.779 |
| *C. carpio* | UAIC_50866.1 | UAIC 50866.01 | *C. cyprinus* | *C. carpio* | PV579758 | PV579825 | Rio Grande | 29.32732 | -103.55365 |
| *C. carpio* | UAIC_50866.2 | UAIC 50866.01 | *C. cyprinus* | *C. carpio* | PV579759 | PV579826 | Rio Grande | 29.32732 | -103.55365 |
| *C. carpio* | UAIC_50867.1 | UAIC 50867.01 | - | - | PV579760 | PV579827 | Rio Grande | 35.17141 | -105.04985 |
| *C. carpio* | UAIC_50867.2 | UAIC 50867.01 | - | - | PV579761 | PV579828 | Rio Grande | 35.17141 | -105.04985 |
| *C. carpio* | TU 210.01 | TU 196730 | - | - | JN053221 | - | Sabine | 32.22454 | -94.22665 |
| *C. carpio* | TU 257.01 | Photo Voucher | - | - | JN053260 | - | Sabine | 30.84501 | -93.56738 |
| *C. carpio* | LSRE_1A2 | - | - | - | PV579694 | PV579769 | Sabine | 32.06214 | -94.1883 |
| *C. carpio* | MSRE_1B4 | - | - | - | PV579695 | PV579770 | Sabine | 32.41727 | -94.7093 |
| *C. carpio* | MSRE_1B5 | - | - | - | PV579696 | PV579771 | Sabine | 32.41727 | -94.7093 |
| *C. carpio* | MSRE_1B6 | - | - | - | PV579697 | PV579772 | Sabine | 32.41727 | -94.7093 |
| *C. carpio* | MSRE_2A8 | - | - | - | PV579698 | PV579773 | Sabine | 32.32826 | -94.3538 |
| *C. carpio* | MSRE_2B1 | - | - | - | PV579699 | PV579774 | Sabine | 32.32826 | -94.3538 |
| *C. carpio* | TU 255.01 | TU 202119 | - | - | JN053258 | - | Trinity | 30.2779 | -94.7991 |
| *C.* *cyprinus* | TU 214.04 | TU 198217 | *C.* cf. *cyprinus* | *C.* *cyprinus* | JN053229 | - | Apalachicola | 30.60967 | -84.93044 |
| *C. cyprinus* | TU 214.08 | TU 198217 | *C.* cf. *cyprinus* | *C. cyprinus* | JN053230 | - | Apalachicola | 30.60967 | -84.93044 |
| *C.* *cyprinus* | NCSM_53275 | NCSM 53275 | *C.* cf. *cyprinus* | *C.* *cyprinus* | PV579723 | PV579794 | Apalachicola | 30.7076 | -84.8626 |
| *C.* *cyprinus* | 2015-0849 | UF 238177 | *-* | *-* | PV579766 | PV579832 | Apalachicola | 30.13243 | -85.13914 |
| *C.* *cyprinus* | 2015-0851 | UF 238177 | *-* | *-* | PV579767 | PV579833 | Apalachicola | 30.13243 | -85.13914 |
| *C.* *cyprinus* | 2015-0854 | UF 238188 | *-* | *-* | PV579768 | PV579834 | Apalachicola | 30.15758 | -85.13316 |
| *C.* *cyprinus* | TU 215.16 | TU 198186 | *C.* cf. *cyprinus* | *C.* *cyprinus* | JN053232 | - | Choctawhatchee | 31.02165 | -85.85779 |
| *C.* *cyprinus* | TU 216.15 | TU 198191 | *C.* cf. *cyprinus* | *C.* *cyprinus* | JN053234 | - | Escambia | 31.06491 | -87.09836 |
| *C.* *cyprinus* | TU 218.12 | TU 198204 | *C.* cf. *cyprinus* | *C.* *cyprinus* | JN053236 | - | Pascagoula | 30.65043 | -88.6367 |
| *C.* *cyprinus* | TU 157.01 | TU 196027 | *C.* cf. *cyprinus* | *C.* *cyprinus* | JN053206 | - | Pearl | 30.5349 | -89.8181 |
| *C.* *cyprinus* | TU 102.05 | TU 194165 | *C.* cf. *cyprinus* | *C.* *cyprinus* | JN053175 | - | Pontchartrain | 30.3692 | -90.938 |
| *C. cyprinus* | TU 213.10 | TU 198183 | *C.* cf. *cyprinus* | *C. cyprinus* | JN053228 | - | Altamaha | 32.81425 | -82.96346 |
| *C. cyprinus* | TU 139.01 | TU 194160 | - | - | JN053199 | - | Great Lakes | 44.453075 | -88.069093 |
| *C. cyprinus* | TU 185.04 | - | - | - | JN053218 | - | Great Lakes | 43.70003 | -79.488262 |
| *C. cyprinus* | ROMI-T00770L | ROMI070947 | - | - | PV579731 | - | Great Lakes | 42.04722 | -83.10667 |
| *C. cyprinus* | ROMI-T01815M | ROMI080259 | - | - | PV579732 | PV579802 | Great Lakes | 46.25806 | -72.82472 |
| *C. cyprinus* | ROMI-T03315M | ROMI086690 | - | - | PV579733 | PV579803 | Great Lakes | 44.0985 | -77.56667 |
| *C. cyprinus* | ROMI-T23570F | ROMI102769 | - | - | PV579734 | - | Great Lakes | 44.965672 | -80.947949 |
| *C. cyprinus* | TU 170.01 | Photo Voucher | - | - | JN053209 | - | Hudson | 50.441271 | -101.318509 |
| *C. cyprinus* | TU 170.02 | Photo Voucher | - | - | JN053210 | - | Hudson | 50.441271 | -101.318509 |
| *C. cyprinus* | TU 172.01 | Photo Voucher | - | - | JN053211 | - | Hudson | 50.292942 | -96.862136 |
| *C. cyprinus* | TU 172.02 | Photo Voucher | - | - | JN053212 | - | Hudson | 50.292942 | -96.862136 |
| *C. cyprinus* | TU 173.02 | Photo Voucher | - | - | JN053213 | - | Hudson | 50.292942 | -96.862136 |
| *C. cyprinus* | TU 173.03 | Photo Voucher | - | - | JN053214 | - | Hudson | 50.292942 | -96.862136 |
| *C. cyprinus* | TU 174.01 | Photo Voucher | - | - | JN053215 | - | Hudson | 50.038835 | -110.734261 |
| *C. cyprinus* | TU 174.02 | Photo Voucher | - | - | JN053216 | - | Hudson | 50.038835 | -110.734261 |
| *C. cyprinus* | TU 175.01 | Photo Voucher | - | - | JN053217 | - | Hudson | 53.205059 | -105.782184 |
| *C. cyprinus* | TU 187.01 | Photo Voucher | - | - | JN053219 | - | Hudson | - | - |
| *C. cyprinus* | TU 189.01 | Photo Voucher | - | - | JN053220 | - | Hudson | 49.697651 | -98.902315 |
| *C. cyprinus* | TU 134.14 | TU 192308 | - | - | JN053196 | - | James | 37.56 | -77.57 |
| *C. cyprinus* | TU 107.25 | TU 185678 | - | - | JN053178 | - | Mississippi | 43.29361 | -89.72028 |
| *C. cyprinus* | TU 107.36 | TU 185678 | - | - | JN053179 | - | Mississippi | 43.29361 | -89.72028 |
| *C. cyprinus* | TU 108.11 | TU 192340 | - | - | JN053183 | - | Mississippi | 37.893988 | -84.261659 |
| *C. cyprinus* | TU 119.10 | TU 195172 | - | - | JN053191 | - | Mississippi | 35.977416 | -87.822416 |
| *C. cyprinus* | TU 124.26 | - | - | - | JN053195 | - | Mississippi | - | - |
| *C. cyprinus* | TU 240.01 | - | - | - | JN053241 | - | Mississippi | 41.11003 | -100.721088 |
| *C. cyprinus* | TU 241.01 | - | - | - | JN053242 | - | Mississippi | 41.839212 | -103.634694 |
| *C. cyprinus* | FMNH_118315 | FMNH 118315 | - | - | PV579702 | PV579777 | Mississippi | 41.39 | -88.2575 |
| *C. cyprinus* | FMNH_118316 | FMNH 118316 | - | - | PV579703 | PV579778 | Mississippi | 41.3019 | -89.0375 |
| *C. cyprinus* | NCSM_36218.1 | NCSM 36218 | - | - | PV579716 | PV579788 | Mississippi | 39.8294 | -91.786 |
| *C. cyprinus* | NCSM_48274.1 | NCSM 48274 | - | - | PV579722 | PV579793 | Mississippi | 35.7936 | -82.7107 |
| *C. cyprinus* | NCSM_74538.1 | NCSM 74538 | - | - | PV579725 | PV579796 | Mississippi | 39.5287 | -81.517 |
| *C. cyprinus* | OSUM_114015 | OSUM 114015 | - | - | PV579726 | PV579797 | Mississippi | 41.0666 | -81.9096 |
| *C. cyprinus* | OSUM_116749 | OSUM 116749 | - | - | PV579730 | PV579801 | Mississippi | 39.4676 | -81.4864 |
| *C. cyprinus* | TU 258.01 | NCSM 29738 | *C.* cf. *cyprinus* | *C. cyprinus* | JN053261 | - | Pee Dee | 35.63611 | -80.27222 |
| *C. cyprinus* | TU 261.01 | NCSM 35950 | - | - | JN053263 | - | Pee Dee | 35.0965 | -79.9086 |
| *C. cyprinus* | NCSM_35950.1 | NCSM 35950 | *C.* cf. *cyprinus* | *C. cyprinus* | PV579715 | PV579787 | Pee Dee | 35.0965 | -79.9086 |
| *C. cyprinus* | NCSM_59974.1 | NCSM 59974 | - | - | PV579724 | PV579795 | Potomac | 38.9318 | -77.1173 |
| *C. cyprinus* | TU 135.05 | TU 192309 | - | - | JN053197 | - | Roanoke | 37.21 | -79.9 |
| *C. cyprinus* | NCSM_45777.1 | NCSM 45777 | - | - | PV579718 | PV579790 | Roanoke | 36.4243 | -77.5751 |
| *C. cyprinus* | UAIC_13462.02 | UAIC 13462.02 | - | - | PV579756 | PV579823 | Roanoke | 37.0456 | -79.84443 |
| *C. cyprinus* | TU 148.12 | TU 196163 | *C.* cf. *cyprinus* | *C. cyprinus* | JN053203 | - | Santee | 33.9651 | -81.03635 |
| *C. cyprinus* | NCSM_32350.1 | NCSM 32350 | *C.* cf. *cyprinus* | *C. cyprinus* | PV579713 | PV579786 | Santee | 34.8259 | -81.4723 |
| *C. cyprinus* | TU 149.01 | TU 196168 | - | - | JN053205 | - | Savannah | 33.30923 | -81.856 |
| *C. cyprinus* | NCSM_46001.1 | NCSM 46001 | - | - | PV579720 | PV579791 | Savannah | 33.3033 | -81.8815 |
| LRCS | TU 146.03 | TU 198401 | *C.* cf. *cyprinus* | LRCS | JN053202 | - | Colorado | 30.49226 | -99.75655 |
| LRCS | TU 242.01 | TU 198406 | *C.* cf. *cyprinus* | LRCS | JN053243 | - | Colorado | 30.49226 | -99.75655 |
| LRCS | TU 242.02 | TU 198406 | *C.* cf. *cyprinus* | LRCS | JN053244 | - | Colorado | 30.49226 | -99.75655 |
| LRCS | 0252Y | TCWC 20599.01 | - | - | PV579174 | PV579434 | Colorado | 30.70334 | -98.95377 |
| LRCS | 0253Y | TCWC 20599.01 | - | - | PV579175 | PV579435 | Colorado | 30.70334 | -98.95377 |
| LRCS | 0254Y | TCWC 20599.01 | - | - | PV579176 | PV579436 | Colorado | 30.70334 | -98.95377 |
| LRCS | 0511Y | TCWC 20599.01 | - | - | PV579177 | PV579437 | Colorado | 30.70334 | -98.95377 |
| LRCS | 0512Y | TCWC 20599.01 | - | - | PV579178 | PV579438 | Colorado | 30.70334 | -98.95377 |
| LRCS | 0513Y | TCWC 20599.01 | - | - | PV579179 | PV579439 | Colorado | 30.70334 | -98.95377 |
| LRCS | 0514Y | TCWC 20599.01 | - | - | PV579180 | PV579440 | Colorado | 30.70334 | -98.95377 |
| LRCS | 0516Y | TCWC 20599.01 | - | - | PV579181 | PV579441 | Colorado | 30.70334 | -98.95377 |
| LRCS | 0519Y | TCWC 20599.01 | - | - | PV579182 | PV579442 | Colorado | 30.70334 | -98.95377 |
| LRCS | 0520Y | TCWC 20599.01 | - | - | PV579183 | PV579443 | Colorado | 30.70334 | -98.95377 |
| LRCS | 0521Y | TCWC 20599.01 | - | - | PV579184 | PV579444 | Colorado | 30.70334 | -98.95377 |
| LRCS | 0522Y | TCWC 20599.01 | - | - | PV579185 | PV579445 | Colorado | 30.70334 | -98.95377 |
| LRCS | 0523Y | TCWC 20599.01 | - | - | PV579186 | PV579446 | Colorado | 30.70334 | -98.95377 |
| LRCS | 0525Y | TCWC 20599.01 | - | - | PV579187 | PV579447 | Colorado | 30.70334 | -98.95377 |
| LRCS | 0256Y | TCWC 20600.01 | - | - | PV579188 | PV579448 | Colorado | 30.6381601 | -98.468331 |
| LRCS | 0255Y | TCWC 20600.02 | - | - | PV579189 | PV579449 | Colorado | 30.6381601 | -98.468331 |
| LRCS | 0258Y | TCWC 20601.02 | - | - | PV579194 | PV579454 | Colorado | 30.77785 | -98.5625 |
| LRCS | 0260Y | TCWC 20601.02 | - | - | PV579195 | PV579455 | Colorado | 30.77785 | -98.5625 |
| LRCS | 0262Y | TCWC 20601.02 | - | - | PV579196 | PV579456 | Colorado | 30.77785 | -98.5625 |
| LRCS | 0264Y | TCWC 20601.02 | - | - | PV579197 | PV579457 | Colorado | 30.77785 | -98.5625 |
| LRCS | 0265Y | TCWC 20601.02 | - | - | PV579198 | PV579458 | Colorado | 30.77785 | -98.5625 |
| LRCS | 0267Y | TCWC 20601.02 | - | - | PV579199 | PV579459 | Colorado | 30.77785 | -98.5625 |
| LRCS | 0268Y | TCWC 20601.02 | - | - | PV579200 | PV579460 | Colorado | 30.77785 | -98.5625 |
| LRCS | 0269Y | TCWC 20601.02 | - | - | PV579201 | PV579461 | Colorado | 30.77785 | -98.5625 |
| LRCS | 0271Y | TCWC 20602.01 | - | - | PV579202 | PV579462 | Colorado | 30.7477 | -98.72094 |
| LRCS | 0272Y | TCWC 20602.01 | - | - | PV579203 | PV579463 | Colorado | 30.7477 | -98.72094 |
| LRCS | 0273Y | TCWC 20602.01 | - | - | PV579204 | PV579464 | Colorado | 30.7477 | -98.72094 |
| LRCS | 0274Y | TCWC 20602.01 | - | - | PV579205 | PV579465 | Colorado | 30.7477 | -98.72094 |
| LRCS | 0275Y | TCWC 20602.01 | - | - | PV579206 | PV579466 | Colorado | 30.7477 | -98.72094 |
| LRCS | 0294Y | TCWC 20602.01 | - | - | PV579207 | PV579467 | Colorado | 30.7477 | -98.72094 |
| LRCS | 0295Y | TCWC 20602.01 | - | - | PV579208 | PV579468 | Colorado | 30.7477 | -98.72094 |
| LRCS | 0297Y | TCWC 20602.01 | - | - | PV579209 | PV579469 | Colorado | 30.7477 | -98.72094 |
| LRCS | 0298Y | TCWC 20602.01 | - | - | PV579210 | PV579470 | Colorado | 30.7477 | -98.72094 |
| LRCS | 0300Y | TCWC 20602.01 | - | - | PV579211 | PV579471 | Colorado | 30.7477 | -98.72094 |
| LRCS | 0281Y | TCWC 20603.01 | - | - | PV579212 | PV579472 | Colorado | 30.71156 | -98.88468 |
| LRCS | 0282Y | TCWC 20603.01 | - | - | PV579213 | PV579473 | Colorado | 30.71156 | -98.88468 |
| LRCS | 0284Y | TCWC 20603.01 | - | - | PV579214 | PV579474 | Colorado | 30.71156 | -98.88468 |
| LRCS | 0285Y | TCWC 20603.01 | - | - | PV579215 | PV579475 | Colorado | 30.71156 | -98.88468 |
| LRCS | 0287Y | TCWC 20603.01 | - | - | PV579216 | PV579476 | Colorado | 30.71156 | -98.88468 |
| LRCS | 0289Y | TCWC 20603.01 | - | - | PV579217 | PV579477 | Colorado | 30.71156 | -98.88468 |
| LRCS | 0290Y | TCWC 20603.01 | - | - | PV579218 | PV579478 | Colorado | 30.71156 | -98.88468 |
| LRCS | 0291Y | TCWC 20603.01 | - | - | PV579219 | PV579479 | Colorado | 30.71156 | -98.88468 |
| LRCS | 0292Y | TCWC 20603.01 | - | - | PV579220 | PV579480 | Colorado | 30.71156 | -98.88468 |
| LRCS | 0293Y | TCWC 20603.01 | - | - | PV579221 | PV579481 | Colorado | 30.71156 | -98.88468 |
| LRCS | 0276Y | TCWC 20604.01 | - | - | PV579222 | PV579482 | Colorado | 30.67951 | -99.02734 |
| LRCS | 0277Y | TCWC 20604.01 | - | - | PV579223 | PV579483 | Colorado | 30.67951 | -99.02734 |
| LRCS | 0278Y | TCWC 20604.01 | - | - | PV579224 | PV579484 | Colorado | 30.67951 | -99.02734 |
| LRCS | 0279Y | TCWC 20604.01 | - | - | PV579225 | PV579485 | Colorado | 30.67951 | -99.02734 |
| LRCS | 0280Y | TCWC 20604.01 | - | - | PV579226 | PV579486 | Colorado | 30.67951 | -99.02734 |
| LRCS | 0932Y | TCWC 20604.01 | - | - | PV579227 | PV579487 | Colorado | 30.67951 | -99.02734 |
| LRCS | 0933Y | TCWC 20604.01 | - | - | PV579228 | PV579488 | Colorado | 30.67951 | -99.02734 |
| LRCS | 0934Y | TCWC 20605.01 | - | - | PV579229 | PV579489 | Colorado | 30.5893 | -99.59476 |
| LRCS | 0935Y | TCWC 20605.01 | - | - | PV579230 | PV579490 | Colorado | 30.5893 | -99.59476 |
| LRCS | 0936Y | TCWC 20605.01 | - | - | PV579231 | PV579491 | Colorado | 30.5893 | -99.59476 |
| LRCS | 0937Y | TCWC 20605.01 | - | - | PV579232 | PV579492 | Colorado | 30.5893 | -99.59476 |
| LRCS | 0940Y | TCWC 20605.01 | - | - | PV579233 | PV579493 | Colorado | 30.5893 | -99.59476 |
| LRCS | 0941Y | TCWC 20606.01 | - | - | PV579234 | PV579494 | Colorado | 31.00472 | -99.26817 |
| LRCS | 0942Y | TCWC 20606.01 | - | - | PV579235 | PV579495 | Colorado | 31.00472 | -99.26817 |
| LRCS | 0943Y | TCWC 20606.01 | - | - | PV579236 | PV579496 | Colorado | 31.00472 | -99.26817 |
| LRCS | 0944Y | TCWC 20606.01 | - | - | PV579237 | PV579497 | Colorado | 31.00472 | -99.26817 |
| LRCS | 0946Y | TCWC 20606.01 | - | - | PV579238 | PV579498 | Colorado | 31.00472 | -99.26817 |
| LRCS | 0947Y | TCWC 20606.01 | - | - | PV579239 | PV579499 | Colorado | 31.00472 | -99.26817 |
| LRCS | 0948Y | TCWC 20606.01 | - | - | PV579240 | PV579500 | Colorado | 31.00472 | -99.26817 |
| LRCS | 0949Y | TCWC 20606.01 | - | - | PV579241 | PV579501 | Colorado | 31.00472 | -99.26817 |
| LRCS | 0950Y | TCWC 20606.01 | - | - | PV579242 | PV579502 | Colorado | 31.00472 | -99.26817 |
| LRCS | 0945Y | TCWC 20606.02 | - | - | PV579243 | PV579503 | Colorado | 31.00472 | -99.26817 |
| LRCS | 0170Y | TCWC 20608.02 | - | - | PV579246 | PV579506 | Colorado | 30.91668 | -99.49983 |
| LRCS | 0171Y | TCWC 20608.02 | - | - | PV579247 | PV579507 | Colorado | 30.91668 | -99.49983 |
| LRCS | 0173Y | TCWC 20608.02 | - | - | PV579248 | PV579508 | Colorado | 30.91668 | -99.49983 |
| LRCS | 0168Y | TCWC 20609.01 | - | - | PV579249 | PV579509 | Colorado | 31.18919 | -98.90319 |
| LRCS | 0169Y | TCWC 20609.01 | - | - | PV579250 | PV579510 | Colorado | 31.18919 | -98.90319 |
| LRCS | 0166Y | TCWC 20610.02 | - | - | PV579252 | PV579512 | Colorado | 31.01509 | -99.20449 |
| LRCS | 0625F | TCWC 20611.01 | - | - | PV579253 | PV579513 | Colorado | 31.51745 | -98.74094 |
| LRCS | 0754F | TCWC 20614.02 | - | - | PV579277 | PV579537 | Colorado | 31.0938 | -98.4705 |
| LRCS | 0757F | TCWC 20614.02 | - | - | PV579278 | PV579538 | Colorado | 31.0938 | -98.4705 |
| LRCS | 0768F | TCWC 20616.02 | - | - | PV579286 | PV579546 | Colorado | 31.52156 | -100.09285 |
| LRCS | 0006F | TCWC 20620.02 | - | - | PV579315 | PV579575 | Colorado | 30.27266 | -98.5582 |
| LRCS | 0008F | TCWC 20620.02 | - | - | PV579316 | PV579576 | Colorado | 30.27266 | -98.5582 |
| LRCS | 0011F | TCWC 20620.02 | - | - | PV579317 | PV579577 | Colorado | 30.27266 | -98.5582 |
| LRCS | 0012F | TCWC 20620.02 | - | - | PV579318 | PV579578 | Colorado | 30.27266 | -98.5582 |
| LRCS | 0013F | TCWC 20620.02 | - | - | PV579319 | PV579579 | Colorado | 30.27266 | -98.5582 |
| LRCS | 0015F | TCWC 20620.02 | - | - | PV579320 | PV579580 | Colorado | 30.27266 | -98.5582 |
| LRCS | 0016F | TCWC 20620.02 | - | - | PV579321 | PV579581 | Colorado | 30.27266 | -98.5582 |
| LRCS | 0017F | TCWC 20620.02 | - | - | PV579322 | PV579582 | Colorado | 30.27266 | -98.5582 |
| LRCS | 0018F | TCWC 20620.02 | - | - | PV579323 | PV579583 | Colorado | 30.27266 | -98.5582 |
| LRCS | 0019F | TCWC 20620.02 | - | - | PV579324 | PV579584 | Colorado | 30.27266 | -98.5582 |
| LRCS | 0002F | TCWC 20621.01 | - | - | PV579325 | PV579585 | Colorado | 30.32181 | -98.44191 |
| LRCS | 0003F | TCWC 20621.01 | - | - | PV579326 | PV579586 | Colorado | 30.32181 | -98.44191 |
| LRCS | 0640F | TCWC 20621.01 | - | - | PV579327 | PV579587 | Colorado | 30.32181 | -98.44191 |
| LRCS | 0641F | TCWC 20621.01 | - | - | PV579328 | PV579588 | Colorado | 30.32181 | -98.44191 |
| LRCS | 0642F | TCWC 20621.01 | - | - | PV579329 | PV579589 | Colorado | 30.32181 | -98.44191 |
| LRCS | 0647F | TCWC 20621.01 | - | - | PV579330 | PV579590 | Colorado | 30.32181 | -98.44191 |
| LRCS | 0648F | TCWC 20621.01 | - | - | PV579331 | PV579591 | Colorado | 30.32181 | -98.44191 |
| LRCS | 0649F | TCWC 20621.01 | - | - | PV579332 | PV579592 | Colorado | 30.32181 | -98.44191 |
| LRCS | 0650F | TCWC 20621.01 | - | - | PV579333 | PV579593 | Colorado | 30.32181 | -98.44191 |
| LRCS | 0004F | TCWC 20621.02 | - | - | PV579334 | PV579594 | Colorado | 30.32181 | -98.44191 |
| LRCS | 0644F | TCWC 20621.02 | - | - | PV579335 | PV579595 | Colorado | 30.32181 | -98.44191 |
| LRCS | 0035F | TCWC 20622.02 | - | - | PV579340 | PV579600 | Colorado | 30.28636 | -98.38299 |
| LRCS | 0037F | TCWC 20622.02 | - | - | PV579341 | PV579601 | Colorado | 30.28636 | -98.38299 |
| LRCS | 0042F | TCWC 20622.02 | - | - | PV579342 | PV579602 | Colorado | 30.28636 | -98.38299 |
| LRCS | 0045F | TCWC 20622.02 | - | - | PV579343 | PV579603 | Colorado | 30.28636 | -98.38299 |
| LRCS | 0031F | TCWC 20622.03 | - | - | PV579344 | PV579604 | Colorado | 30.28636 | -98.38299 |
| LRCS | 0047F | TCWC 20622.03 | - | - | PV579345 | PV579605 | Colorado | 30.28636 | -98.38299 |
| LRCS | 0626F | TCWC 20622.03 | - | - | PV579346 | PV579606 | Colorado | 30.28636 | -98.38299 |
| LRCS | 0636F | TCWC 20622.03 | - | - | PV579347 | PV579607 | Colorado | 30.28636 | -98.38299 |
| LRCS | 0026F | TCWC 20623.02 | - | - | PV579350 | PV579610 | Colorado | 30.3342 | -98.24779 |
| LRCS | 0028F | TCWC 20623.02 | - | - | PV579351 | PV579611 | Colorado | 30.3342 | -98.24779 |
| LRCS | 0027F | TCWC 20623.03 | - | - | PV579352 | PV579612 | Colorado | 30.3342 | -98.24779 |
| LRCS | 0804F | TCWC 20623.03 | - | - | PV579353 | PV579613 | Colorado | 30.3342 | -98.24779 |
| LRCS | 0805F | TCWC 20623.03 | - | - | PV579354 | PV579614 | Colorado | 30.3342 | -98.24779 |
| LRCS | 0811F | TCWC 20624.02 | - | - | PV579358 | PV579618 | Colorado | 30.3358 | -98.13906 |
| LRCS | 0816F | TCWC 20624.02 | - | - | PV579359 | PV579619 | Colorado | 30.3358 | -98.13906 |
| LRCS | 0822F | TCWC 20625.02 | - | - | PV579362 | PV579622 | Colorado | 30.36237 | -98.12634 |
| LRCS | 0823F | TCWC 20625.02 | - | - | PV579363 | PV579623 | Colorado | 30.36237 | -98.12634 |
| LRCS | 0093F | TCWC 20626.01 | - | - | PV579364 | PV579624 | Colorado | 30.65433 | -99.32143 |
| LRCS | 0094F | TCWC 20626.01 | - | - | PV579365 | PV579625 | Colorado | 30.65433 | -99.32143 |
| LRCS | 0095F | TCWC 20626.01 | - | - | PV579366 | PV579626 | Colorado | 30.65433 | -99.32143 |
| LRCS | 0096F | TCWC 20626.01 | - | - | PV579367 | PV579627 | Colorado | 30.65433 | -99.32143 |
| LRCS | 0097F | TCWC 20626.01 | - | - | PV579368 | PV579628 | Colorado | 30.65433 | -99.32143 |
| LRCS | 0098F | TCWC 20626.01 | - | - | PV579369 | PV579629 | Colorado | 30.65433 | -99.32143 |
| LRCS | 0099F | TCWC 20626.01 | - | - | PV579370 | PV579630 | Colorado | 30.65433 | -99.32143 |
| LRCS | 0100F | TCWC 20626.01 | - | - | PV579371 | PV579631 | Colorado | 30.65433 | -99.32143 |
| LRCS | 0824F | TCWC 20626.01 | - | - | PV579372 | PV579632 | Colorado | 30.65433 | -99.32143 |
| LRCS | 0825F | TCWC 20626.01 | - | - | PV579373 | PV579633 | Colorado | 30.65433 | -99.32143 |
| LRCS | 0084F | TCWC 20629.02 | - | - | PV579382 | PV579642 | Colorado | 30.01293 | -97.16016 |
| LRCS | 0085F | TCWC 20629.02 | - | - | PV579383 | PV579643 | Colorado | 30.01293 | -97.16016 |
| LRCS | 0086F | TCWC 20629.02 | - | - | PV579384 | PV579644 | Colorado | 30.01293 | -97.16016 |
| LRCS | 0088F | TCWC 20629.02 | - | - | PV579385 | PV579645 | Colorado | 30.01293 | -97.16016 |
| LRCS | 0077F | TCWC 20630.02 | - | - | PV579392 | PV579652 | Colorado | 29.89238 | -96.87729 |
| LRCS | 0245F | TCWC 20630.02 | - | - | PV579393 | PV579653 | Colorado | 29.89238 | -96.87729 |
| LRCS | 0246F | TCWC 20630.02 | - | - | PV579394 | PV579654 | Colorado | 29.89238 | -96.87729 |
| LRCS | 0247F | TCWC 20630.02 | - | - | PV579395 | PV579655 | Colorado | 29.89238 | -96.87729 |
| LRCS | 0248F | TCWC 20630.02 | - | - | PV579396 | PV579656 | Colorado | 29.89238 | -96.87729 |
| LRCS | 0249F | TCWC 20630.02 | - | - | PV579397 | PV579657 | Colorado | 29.89238 | -96.87729 |
| LRCS | 0250F | TCWC 20630.02 | - | - | PV579398 | PV579658 | Colorado | 29.89238 | -96.87729 |
| LRCS | 0243F | TCWC 20630.03 | - | - | PV579399 | PV579659 | Colorado | 29.89238 | -96.87729 |
| LRCS | 0226F | TCWC 20633.01 | - | - | PV579406 | PV579666 | Colorado | 30.5372 | -98.17579 |
| LRCS | 0228F | TCWC 20633.01 | - | - | PV579407 | PV579667 | Colorado | 30.5372 | -98.17579 |
| LRCS | TNHC_424 | TNHC 50107 | *C. cyprinus* | LRCS | PV579747 | PV579813 | Colorado | 30.2226 | -97.40947 |
| LRCS | 0198F | TCWC 20805.01 | - | - | PV579431 | PV579691 | Guadalupe | 29.99711 | -99.10577 |
| LRCS | 0200F | TCWC 20805.01 | - | - | PV579432 | PV579692 | Guadalupe | 29.99711 | -99.10577 |
| LRCS | 0899F | TCWC 20805.01 | - | - | PV579433 | PV579693 | Guadalupe | 29.99711 | -99.10577 |
| LRCS | TNHC_2220 | TNHC 69839 | - | - | PV579754 | PV579820 | San Bernard | 29.77977 | -96.31509 |
| LRCS | TNHC_2222 | TNHC 69839 | - | - | PV579755 | PV579821 | San Bernard | 29.77977 | -96.31509 |
| *C. velifer* | TU 213.01 | TU 198184 | *C.* cf. *velifer* | *C. velifer* | JN053224 | - | Altamaha | 32.81425 | -82.96346 |
| *C. velifer* | TU 213.04 | TU 198184 | *C.* cf. *velifer* | *C. velifer* | JN053225 | - | Altamaha | 32.81425 | -82.96346 |
| *C. velifer* | TU 213.05 | TU 198184 | *C.* cf. *velifer* | *C. velifer* | JN053226 | - | Altamaha | 32.81425 | -82.96346 |
| *C. velifer* | TU 213.07 | TU 198184 | *C.* cf. *velifer* | *C. velifer* | JN053227 | - | Altamaha | 32.81425 | -82.96346 |
| *C. velifer* | TU 271.02 | NCSM 45842 | *C.* cf. *velifer* | *C. velifer* | JN053265 | - | Apalachicola | 30.703 | -84.8602 |
| *C. velifer* | NCSM_45841.1 | NCSM 45841 | *C.* cf. *velifer* | *C. velifer* | PV579719 | - | Apalachicola | 30.703 | -84.8602 |
| *C. velifer* | TU 215.06 | TU 198188 | *C.* cf. *velifer* | *C. velifer* | JN053231 | - | Choctawhatchee | 31.02165 | -85.85779 |
| *C. velifer* | TU 216.06 | TU 198193 | *C.* cf. *velifer* | *C. velifer* | JN053233 | - | Escambia | 31.06491 | -87.09836 |
| *C. velifer* | 2015-0823 | UF 238170 | - | - | PV579764 | - | Escambia | 30.66988 | -87.26618 |
| *C. velifer* | 2015-0825 | UF 238170 | - | - | PV579765 | PV579831 | Escambia | 30.66988 | -87.26618 |
| *C. velifer* | TU 107.42 | TU 185679 | - | - | JN053180 | - | Mississippi | 43.29361 | -89.72028 |
| *C. velifer* | TU 107.45 | TU 185679 | - | - | JN053181 | - | Mississippi | 43.29361 | -89.72028 |
| *C. velifer* | TU 107.50 | TU 185679 | - | - | JN053182 | - | Mississippi | 43.29361 | -89.72028 |
| *C. velifer* | TU 108.16 | TU 192341 | - | - | JN053184 | JX488939 | Mississippi | 37.893988 | -84.261659 |
| *C. velifer* | TU 116.27 | TU 194142 | - | - | JN053186 | - | Mississippi | 43.29361 | -89.72028 |
| *C. velifer* | TU 116.24 | TU 194142 | - | - | JN053189 | - | Mississippi | 43.29361 | -89.72028 |
| *C. velifer* | TU 119.13 | TU 195173 | - | - | JN053192 | - | Mississippi | 35.977416 | -87.822416 |
| *C. velifer* | FMNH_137556 | FMNH 137556 | - | - | PV579704 | PV579779 | Mississippi | 41.355563 | -88.207072 |
| *C. velifer* | INHS_110038 | INHS 110038 | - | - | PV579705 | - | Mississippi | 41.208568 | -88.93034 |
| *C. velifer* | OSUM_116739 | OSUM 116739 | - | - | PV579727 | PV579798 | Mississippi | 38.9695 | -83.0423 |
| *C. velifer* | OSUM_116747 | OSUM 116747 | - | - | PV579728 | PV579799 | Mississippi | 39.4689 | -81.4914 |
| *C. velifer* | TU 217.09 | TU 198197 | *C.* cf. *velifer* | *C. velifer* | JN053235 | - | Mobil | 32.66832 | -87.24136 |
| *C. velifer* | UAIC_49654.01 | UAIC 49654.01 | - | - | PV579757 | PV579824 | Mobil | 32.66833 | -87.24195 |
| *C. velifer* | TU 141.01 | TU 195402 | *C.* cf. *velifer* | *C. velifer* | JN053200 | - | Pascagoula | 30.65043 | -88.6367 |
| *C. velifer* | TU 157.07 | TU 196054 | *C.* cf. *velifer* | *C. velifer* | JN053207 | - | Pearl | 30.5349 | -89.8181 |
| *C. velifer* | TU 103.37 | TU 196161 | *C.* cf. *velifer* | *C. velifer* | JN053176 | - | Pontchartrain | 30.43468 | -90.7734 |
| *C. velifer* | TU 148.26 | TU 196164 | *C.* cf. *velifer* | *C. velifer* | JN053204 | - | Santee | 33.9651 | -81.03635 |
| *C. velifer* | NCSM_32336.1 | NCSM 32336 | *C.* cf. *velifer* | *C. velifer* | PV579712 | - | Santee | 33.918 | -81.0249 |
| *C. velifer* | NCSM_32351.1 | NCSM 32351 | *C.* cf. *velifer* | *C. velifer* | PV579714 | - | Santee | 34.8259 | -81.4723 |
| *C. velifer* | TU 136.04 | - | *C.* cf. *velifer* | *C. velifer* | JN053198 | - | Savannah |  |  |
| *C. velifer* | TU 239.01 | Photo Voucher | *C.* cf. *velifer* | *C. velifer* | JN053240 | - | Savannah | 32.553217 | -81.279067 |

Table S2. Summary of tissues and specimens available for analysis compiled across this study. All tissues from field collections were successfully extracted and amplified for mitochondrial cytochrome b (CYTB) and nuclear interphotoreceptor retinoid-binding protein 2 (IRBP2) genes leading to 266 specimens available for analysis for CYTB and IRBP2. Of the 72 requested tissues, 70 extractions were successful for CYTB sequencing. An additional tissue was successfully extracted and sequenced for CYTB (NSCM 45941) but not further analyzed

as it was sequenced following preparation of this study for publication. This led to 69 specimens with available sequences for CYTB analysis from our requested tissues. Regarding IRBP2, 60 extractions were successful from requested tissues. However, one specimen (UAIC 12152.01) was not further analyzed as we were unable to sequence CYTB successfully, leading to 59 specimens available for IRBP2 sequence analysis. These discrepancies, along with different numbers of sequences and associated specimens deposited to GenBank led to the total sample sizes we report below.

| Group | Tissues | Specimens Analyzed (CYTB) | Specimens Analyzed (IRBP2) |
| --- | --- | --- | --- |
| Field Collections | 266 | 266 | 266 |
| Requested Tissues | 72 | 69 | 59 |
| GenBank | - | 90 | 2 |
| Total | 338 | 425 | 327 |
|  |  |  |  |

# **Further Reading:**

*Further Reading - 1*

**Collection Methods-** At each site (Figure A2), a combination of active and passive capture techniques were used to collect *Carpiodes*, including gill nets, seines, and several electrofishing methods. For gill nets, two 36 m long by 1.8 m deep (10-38 mm mesh) monofilament gill nets were set for one hour, optimizing pool habitats with water depths greater than 2 m. For seining, we targeted shallow (<1.5 m depth) stream sections using a 4.6 m by 1.8 m (5 mm mesh) seine. A total of 10 seine hauls of 10 m length were conducted in habitats most likely to produce *Carpiodes* including runs, shallow pools, and riverbanks as done by other studies (Baker et al., 1991; Gelwick and Morgan, 2000; Li and Gelwick, 2003). For electrofishing, either backpack, canoe, or boat deployed methods were implemented at each site depending on water depth and river navigability. In narrow (<10 m) wadable streams (<1.5 m depth) backpack electrofishing was conducted using a Midwest Lakes Electrofishing Systems (MLES) Infinity Xstream electrofishing unit powered by a Lithium smart 24-volt (19.2 amp-hour) battery. In areas with wider wetted widths (>10 m) with similar depths, canoe electrofishing was deployed using a Smith-Root Model 1.5-KVA electrofishing control box powered by a Honda EU2200i generator floated in a 5.2 m canoe. Boat electrofishing was used in reservoirs where water depths exceeded 1.5 m using a 4.8-m aluminum boat with a Midwest Lake Electrofishing Infinity HC-80 control box powered by a Honda EG 4000 CL generator. A total of 900 seconds of electrofishing was conducted regardless of electrofishing method.

*Specimen and Tissue Collection***-** Upon successful collection of *Carpiodes*, fish were placed in a container holding a solution of clove oil at a lethal concentration of 80 mg per liter of water to euthanize specimens before tissue collection (Peake, 1998). Each euthanized fish received a FLOY tag with a unique identifier inserted directly posterior to the dorsal fin on the right side of the fish’s body. Following tagging, a small segment of gill filament tissue (< 6 mm length) from the right side of the fish’s body was removed and placed into a cryo-tube labeled with the tag identification number containing 100% non-denatured ethanol. Each specimen was then injected with an undiluted formalin solution and placed in a container of formalin that was 10% of the injection concentration for storage and transportation back to the laboratory. Specimens were deposited at the Biodiversity Research and Teaching Collections (BRTC) at Texas A&M University. Information on accessioned specimens can be found in (Table S1).

*Further Reading - 2*

Between our subsetted mitochondrial cytochrome b (CYTB) and concatenated trees, topology of across clades did not show major differences other than the position of a clade possessing *Carpiodes velifer* from Eastern Gulf of Mexico (i.e., Eastern Gulf) drainages. In the CYTB subsetted tree (Figure S1a), this clade was sister to all *Carpiodes* lineages other than a clade possessing divergent *Carpiodes cyprinus* from the Eastern Gulf basin (i.e., Apalachicola River). In the subsetted Concatenated tree (Figure S1b), this clade was sister to an additional *C. velifer* clade possessing Mississippi basin specimens united by a polytomy with three *Carpiodes carpio* clades from Mississippi and Western Gulf of Mexico (i.e., Western Gulf) basins.

In Figure S1a, the clade containing Llano River Carpsucker (LRCS), *C. cyprinus,* and *C. carpio* was sister to all *Carpiodes* clades excluding Eastern Gulf *C. cyprinus* and *C. velifer* clades. In Figure S1b, there was a sister relationship between the clade containing LRCS and all *Carpiodes* clades excluding the Eastern Gulf *C.* *cyprinus* clade. Support for position of this clade was variable. Support was low for the relationship in Figure S1a (PP=0.63), while in the Concatenated tree the relationship was unambiguous (PP=1). However, both trees were missing many lineages of *Carpiodes* that were present in the CYTB full tree (main text - Figure 7). Although the main text tree did not observe these relationships and resolved a polytomy between all clades discussed above other than the Eastern Gulf *C.* *cyprinus* clade, the lack of spatial coverage (i.e., missing lineages) makes these trees less informative. Therefore, we interpret the full CYTB tree in the manuscript main text.

# **References:**

Baker, J. A., Killgore, K. J., & Kasul, R. L. (1991). Aquatic habitats and fish communities in the lower Mississippi River. *Reviews in Aquatic Sciences*, *3*(4), 313-356.

Gelwick, F. P., & Morgan, M. N. (2000). *Microhabitat use and community structure of fishes downstream of the proposed George Parkhouse I and Marvin Nichols I reservoir sites on the Sulphur River, TX*. Department of Wildlife and Fisheries Sciences, Texas A & M University.

Li, R. Y., & Gelwick, F. P. (2003). *Mesohabitat use and community structure of Brazos River fishes in the vicinity of the proposed Allens Creek Reservoir*. Texas Water Resources Institute.

Peake, S. (1998). Sodium bicarbonate and clove oil as potential anesthetics for nonsalmonid fishes. *North American Journal of Fisheries Management*, *18*(4), 919-924.
