# Supplementary figures and images for: The Carpiodes Conundrum: Molecular Hypothesis Testing Informs Conservation Applications for Carpsuckers (Catostomidae: Carpiodes) in Texas and Beyond
Source: Ecol Evol. 2025 Nov 23;15(11):e72543. doi: 10.1002/ece3.72543 (PMC12640881; doi:10.1002/ece3.72543)

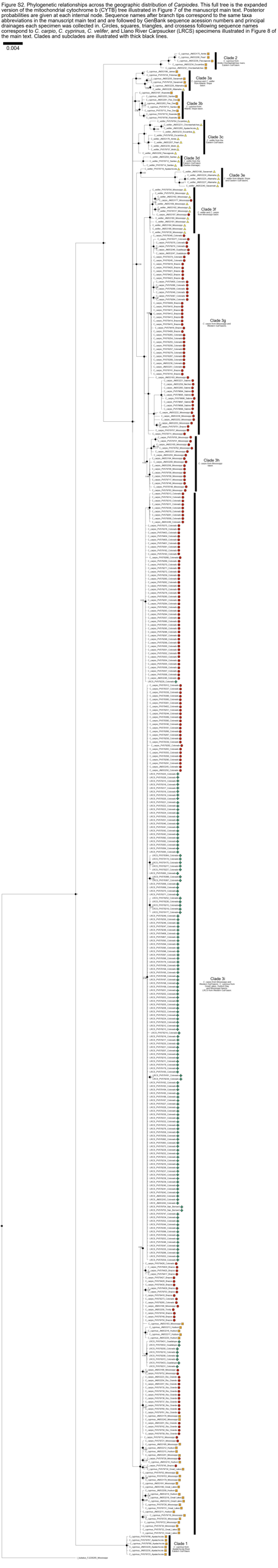

Supplement: Supplementary file 1 — Data S1: ece372543‐sup‐0001‐supinfo.zip. [file ECE3-15-e72543-s001.zip › Figure_S2.jpeg]

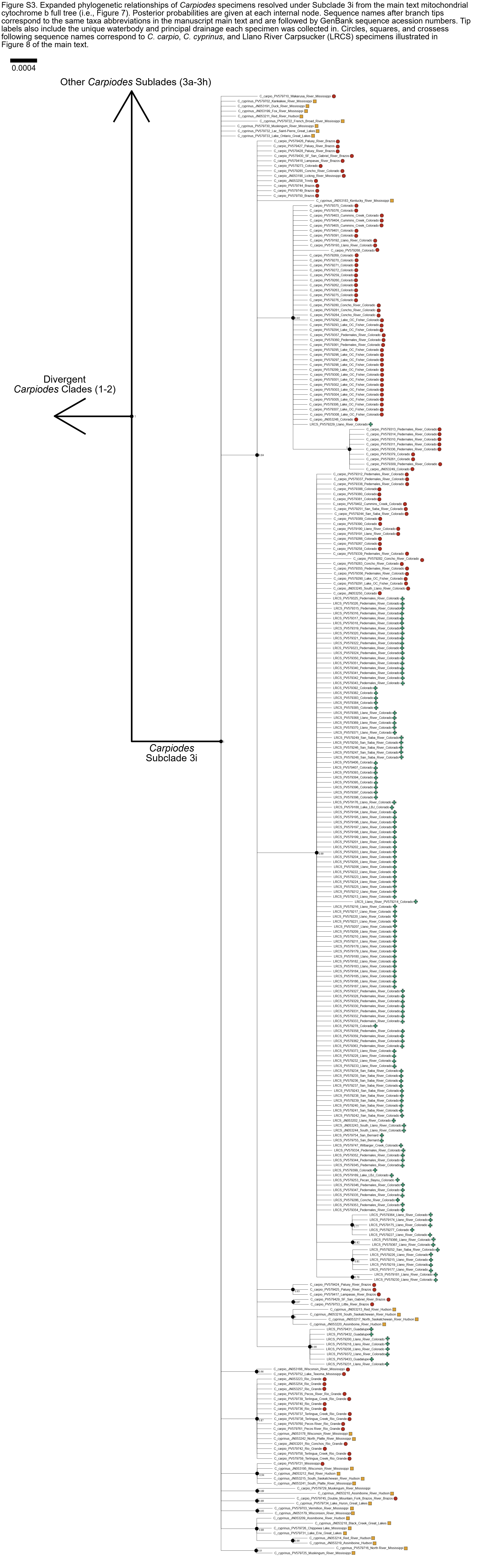

Supplement: Supplementary file 1 — Data S1: ece372543‐sup‐0001‐supinfo.zip. [file ECE3-15-e72543-s001.zip › Figure_S3.jpeg]
